# Deriving and validating a risk prediction model for long COVID: a population-based, retrospective cohort study in Scotland

**DOI:** 10.1177/01410768241297833

**Published:** 2024-11-18

**Authors:** Karen Jeffrey, Vicky Hammersley, Rishma Maini, Anna Crawford, Lana Woolford, Ashleigh Batchelor, David Weatherill, Chris White, Tristan Millington, Robin Kerr, Siddharth Basetti, Calum Macdonald, Jennifer K Quint, Steven Kerr, Syed Ahmar Shah, Amanj Kurdi, Colin R Simpson, Srinivasa Vittal Katikireddi, Igor Rudan, Chris Robertson, Lewis Ritchie, Aziz Sheikh, Luke Daines

**Affiliations:** 1 Usher Institute, University of Edinburgh, Edinburgh EH16 4UX, UK; 2 Public Health Scotland, Glasgow and Edinburgh, UK; 3 NHS Borders, Melrose, UK; 4 NHS Highland, Inverness, UK; 5 National Heart and Lung Institute, Imperial College London, London, UK; 6 Strathclyde Institute of Pharmacy and Biomedical Science, University of Strathclyde, Glasgow G4 0RE, UK; 7 Department of Clinical Pharmacy, College of Pharmacy, Hawler Medical University, Erbil, Iraq; 8 Al-Kitab University, Kirkuk 36015, Iraq; 9 School of Pharmacy, Sefako Makgatho Health Sciences University, Pretoria, South Africa; 10 School of Health, Wellington Faculty of Health, 8491 Victoria University of Wellington, Wellington, New Zealand; 11 MRC/CSO Social & Public Health Sciences Unit, University of Glasgow, Glasgow, UK; 12 Department of Mathematics and Statistics, 3527 University of Strathclyde, Glasgow, UK; 13 Academic Primary Care, University of Aberdeen, Aberdeen, UK; 14 Institute of Applied Health Sciences, University of Aberdeen, Aberdeen AB24 3FX, UK; 15 Nuffield Department of Primary Care Health Sciences, University of Oxford, Oxford OX2 6GG, UK

**Keywords:** Clinical, epidemiologic studies, epidemiology, health informatics, infectious diseases

## Abstract

**Objectives:**

Using electronic health records, we derived and internally validated a prediction model to estimate risk factors for long COVID and predict individual risk of developing long COVID.

**Design:**

Population-based, retrospective cohort study.

**Setting:**

Scotland.

**Participants:**

Adults (≥18 years) with a positive COVID-19 test, registered with a general medical practice between 1 March 2020 and 20 October 2022.

**Main outcome measures:**

Adjusted odds ratios (aORs) with 95% confidence intervals (CIs) for predictors of long COVID, and patients’ predicted probabilities of developing long COVID.

**Results:**

A total of 68,486 (5.6%) patients were identified as having long COVID. Predictors of long COVID were increasing age (aOR: 3.84; 95% CI: 3.66–4.03 and aOR: 3.66; 95% CI: 3.27–4.09 in first and second splines), increasing body mass index (BMI) (aOR: 3.17; 95% CI: 2.78–3.61 and aOR: 3.09; 95% CI: 2.13–4.49 in first and second splines), severe COVID-19 (aOR: 1.78; 95% CI: 1.72–1.84); female sex (aOR: 1.56; 95% CI: 1.53–1.60), deprivation (most versus least deprived quintile, aOR: 1.40; 95% CI: 1.36–1.44), several existing health conditions. Predictors associated with reduced long COVID risk were testing positive while Delta or Omicron variants were dominant, relative to when the Wild-type variant was dominant (aOR: 0.85; 95% CI: 0.81–0.88 and aOR: 0.64; 95% CI: 0.61–0.67, respectively) having received one or two doses of COVID-19 vaccination, relative to unvaccinated (aOR: 0.90; 95% CI: 0.86–0.95 and aOR: 0.96; 95% CI: 0.93–1.00).

**Conclusions:**

Older age, higher BMI, severe COVID-19 infection, female sex, deprivation and comorbidities were predictors of long COVID. Vaccination against COVID-19 and testing positive while Delta or Omicron variants were dominant predicted reduced risk.

## Introduction

Long COVID is a debilitating multi-system condition estimated to affect more than 10% of patients infected with severe acute respiratory syndrome coronavirus 2 (SARS-CoV-2).^1,2^ Individuals with long COVID experience a range of symptoms, including shortness of breath, chest pain and fatigue. These symptoms can last for months or years, lead to a deterioration in quality of life and limit the ability to carry out everyday tasks.^1,3–5^

The burden posed by long COVID has created a need for prediction models to identify patients at greatest risk. These models could inform preventive public health strategies, improve targeted support for patients and guide participant selection for clinical trials aimed at developing therapeutic interventions. Several long COVID prediction models have been developed, and have identified common risk factors, including increasing age,^6–10^ female sex^6,9,11,12^ and the severity of acute COVID-19 infection.^5,8,11^ However, many of these models identify long COVID cases using survey data,^6,7,11,12^ which may be prone to sampling bias, recall bias or inaccurate self-reporting. Other models use data from electronic heath records (EHRs) for case identification, such as long COVID diagnostic codes^8,9^ or referrals to long COVID clinics.^10^ While this approach mitigates some of the challenges associated with survey data, under-utilisation of diagnostic codes and non-universal access to long COVID clinics^13–15^ likely results in under-identification of long COVID cases. This, in turn, restricts the number of positive cases available for model training and increases the likelihood of undocumented cases being misclassified as controls.

Our study sought to address these limitations by taking a multi-faceted approach to case identification. We identified long COVID cases using clinical codes and free-text data from EHRs together with an operational definition for long COVID.^13^ In doing so, we aimed to reduce the risk of bias and under-identification, thereby improving case identification for model training, and enhancing the accuracy of predictions by capturing cases that might otherwise go undocumented.

## Methods

### Study design, setting, participants and permissions

The protocol describing this study was published in advance.^16^ We followed the Transparent Reporting of Multivariable Prediction Models (TRIPOD) guidelines (Table S1).^17^

We conducted a retrospective cohort study using routinely collected data contained in EHRs, hosted on the Early Pandemic Evaluation and Enhanced Surveillance of COVID-19 (EAVE II) platform.^18^ The EAVE II platform provided approved researchers with access to pseudonymised EHRs for all individuals registered with general practitioners (GP) in Scotland (98%–99% of the population) during the COVID-19 pandemic.

**Figure 1.**
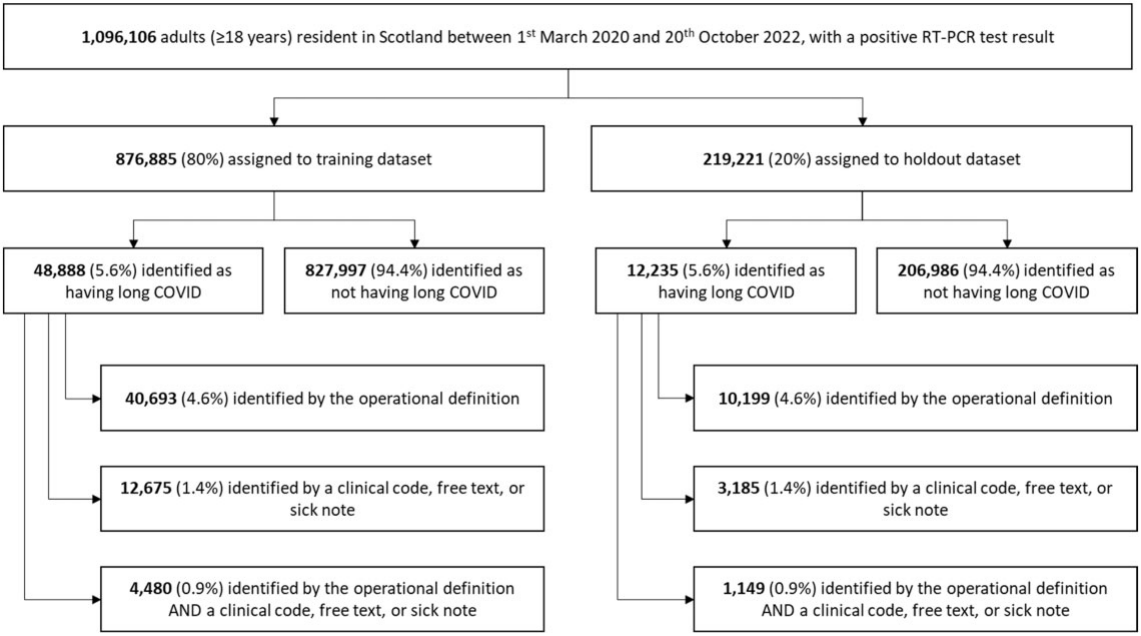
Participants identified as having long COVID in the training and holdout datasets.

We analysed linked data from primary care, secondary care, laboratory testing and prescribing for adults (≥18 years) registered with GPs and resident in Scotland between 1 March 2020 and 20 October 2022 (date of last data extraction). Specific datasets are listed in the Supplementary Materials, pp.S3. The sample was restricted to 1,096,106 individuals infected with SARS-CoV-2 (indicated by a positive reverse transcription polymerase chain reaction (RT-PCR) test result), who had a minimum of four weeks’ follow-up data available after testing (Figure 1).

It was not feasible to obtain consent from each participant; however, the National Health Service of Scotland’s Public Benefit and Privacy Panel for Health and Social Care (PBPP) granted permission to access, within a secure trusted research environment, unconsented, whole-population, de-identified data from EHRs for the purpose of surveillance during a public health emergency.

### Outcome measures

The primary outcome was long COVID. Following a novel case identification approach we recently reported on,^13^ we classified patients as having long COVID if they had one or more of the following in their EHRs: a long COVID clinical code recorded in primary care; free-text terms indicating long COVID recorded in primary care; a sick note containing free-text terms indicating long COVID or patterns in EHR data suggestive of long COVID, as captured by an operational definition.

**Figure 2.**
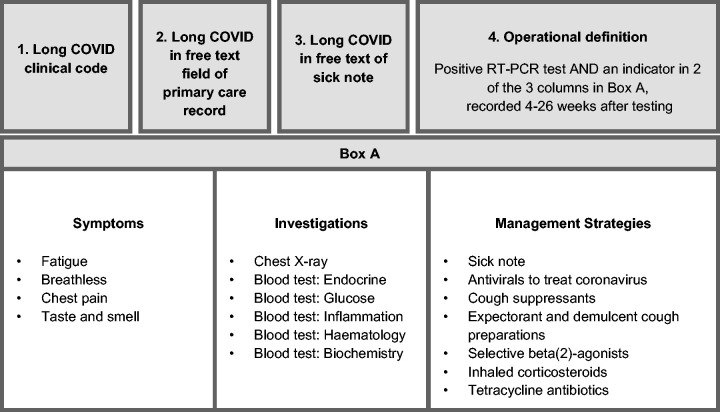
Long COVID outcome measure. Individuals were classified as having long COVID if they had any of the indicators described in boxes 1–4 recorded in their EHR.

The operational definition identified individuals as having long COVID if they had specific combinations of clinical codes and dispensed prescriptions recorded in their EHRs in the 4–26 weeks following a positive RT-PCR test (summarised in Figure 2). The clinical codes and prescriptions included in the operational definition were identified previously, informed by an investigation into clinical interactions that were recorded at a significantly higher rate in the EHRs of individuals who tested positive for COVID-19, relative to matched controls, within 4–26 of each matched pair’s positive RT-PCR test date.

Details on each component of the outcome measure are included in the Supplementary Materials, pp.S4–5.

### Predictors

Candidate predictors were selected based on a review of the literature and assessment by the project’s steering group of clinicians, epidemiologists, data scientists, and patient and public involvement (PPI) members. An index date equal to the date of an individual’s first positive RT-PCR test was used to define time-variant predictors (as described below).

#### Socio-demographic variables

Sex was recorded as a binary variable. We used degree 1 splines in age with knots at 34 and 52 years (two knots optimised the Akaike Information Criterion [AIC] score). Scottish Index of Multiple Deprivation (SIMD) quintiles,^19^ household size and six-fold urban-rural residency were recorded as ordinal variables. Care home residency was recorded as a binary variable.

#### Clinical variables

The number of COVID-19 vaccine doses received up to 14 days before the index date was included as an ordinal variable. We analysed serological data to derive an indicator of the dominant SARS-CoV-2 variant (representing 60% or more of sequenced cases) during the week each individual tested positive. Binary variables captured whether individuals had been advised to shield from COVID-19, were immunocompromised, had a severe acute COVID-19 infection (defined as hospitalisation within 28 days of testing positive for COVID-19) or had been diagnosed with one of 22 comorbidities associated with increased risk of severe COVID-19^20^ (detailed in Table S2). We used degree 1 splines in body mass index (BMI) with a knot at 28 (a single knot in BMI optimised the AIC score).

#### Medications

Binary variables were used to indicate prescriptions dispensed in the community during the three months before the index date. Prescribing data covered 23 categories of prescriptions (Table S3) representing 775 medicinal products, grouped according to British National Formulary (BNF) sub-paragraphs or chemical substances (Table S4). We prioritised medicinal products highlighted in the literature as candidates for preventing or treating long COVID. To avoid circularity, prescriptions included in the operational definition of long COVID were excluded.

### Statistical analyses

R (version 3.6.1) was used for all analyses.

#### Sample size calculation

Sample size calculations were conducted using the R package, pmsampsize.^21^ The minimum required sample size was estimated to be 20,311 with 1133 events, assuming prevalence of the outcome measure of 5.6%, a c-statistic of 0.71 and 56 candidate predictors with 74 candidate predictor parameters (including splines).

#### Training and holdout

Individuals were randomly assigned to a training or holdout dataset in an 80:20 split (Figure 1).

#### Missing data

Missing BMI values were imputed using single imputation by chained equations, with the following variables included in the imputation model: age, sex, ethnicity, urban-rural classification, QCOVID risk groups^22^ and SIMD quintiles. Imputations were carried out separately for men and women over 20 years of age, and those under 20 years of age.

#### Multicollinearity checks

We tested for multicollinearity among predictors using correlation coefficients and variance inflation factors. Type 2 diabetes and prescriptions for metformin (a drug primarily used to treat Type 2 Diabetes) had a correlation coefficient of 0.67. Therefore, a combined ‘Type 2 Diabetes’ measure indicating that a patient had a Type 2 Diabetes clinical code or metformin prescription was used. Variance inflation factors suggested no multicollinearity.

#### Predictor selection

Predictors were selected using backward stepwise selection to optimise the AIC score of the multivariable logistic regression model. This led to the removal of 12 predictors, with no significant impact on model fit (Table S5). Least absolute shrinkage and selection operator regression with resampling was used to assess the robustness of predictor selection, and led to removal of six further predictors, with no significant impact on model performance (Figure S1 and Supplementary Materials, pp.S10).

#### Model type

Multivariable logistic regression with 10-fold cross validation was used for model training.

#### Evaluating model performance

Model performance was evaluated in the training dataset, using the c-statistic, calibration intercept and slope, and by visually inspecting predicted and observed values across vigintiles of predicted probabilities. We considered the true positive and true negative rates to be of equal importance, and therefore evaluated model performance at a discrimination threshold set equal to the prevalence of long COVID observed in the training dataset, using the following performance metrics: sensitivity, specificity, accuracy, positive predicted value (PPV), negative predicted value (NPV), F1 score, Matthew’s correlation coefficient and Brier score.

#### Model validation

To assess the internal validity of the model, we evaluated model performance in the holdout dataset, using the same metrics as for evaluation in the training dataset.

#### Sensitivity analyses

We conducted sensitivity analyses to interrogate the model’s robustness and generalisability, including: using positive lateral flow tests (LFTs), in addition to RT-PCR testing data to identify positive COVID-19 cases for inclusion in the cohort; omitting patients with incomplete follow-up data; using more conservative variations of the main outcome measure; re-training the model using machine learning methods (gradient-boosted decision trees (XGBoost) and Naïve Bayes Classification); and re-training the model using data from 12 of 14 geographic regions in Scotland, and evaluating performance in the two geographic holdout regions (Supplementary Materials, pp.S21-31).

### Patient and public involvement

PPI members were involved in the conception, design, and interpretation of this study (Table S6–7).

## Results

### Participants

The cohort included 1,096,106 adults. There was consistency across training (*n* = 876,885) and holdout (*n* = 219,221) datasets in terms of prevalence of long COVID, (5.6%) participant characteristics, (Table 1) comorbidities and prescriptions (Tables S2–3).

**Table 1.** Patient characteristics in testing and holdout datasets, stratified by long COVID classification.

	Training dataset				Holdout dataset			
	No long COVID		Long COVID		No long COVID		Long COVID	
	*N*	%	*N*	%	*N*	%	*N*	%
Total (% of dataset)	827,997	94.4	48,888	5.6	206,986	94.4	12,235	5.6
*Sex*								
Female	441,891	53.4	31,757	65.0	110,398	53.3	7997	65.4
Male	386,106	46.6	17,131	35.0	96,588	46.7	4238	34.6
*Age (years)*								
18–27	166,790	20.1	4079	8.3	41,605	20.1	987	8.1
28–37	175,219	21.2	6938	14.2	43,641	21.1	1729	14.1
38–47	160,343	19.4	9317	19.1	40,316	19.5	2371	19.4
48–57	145,676	17.6	11,620	23.8	36,536	17.7	2855	23.3
58–67	100,948	12.2	10,186	20.8	25,212	12.2	2541	20.8
68–77	44,267	5.3	4008	8.2	11,110	5.4	1077	8.8
78–87	24,012	2.9	2155	4.4	5886	2.8	525	4.3
88–100	10,742	1.3	585	1.2	2680	1.3	150	1.2
*Scottish Index of Multiple Deprivation (SIMD) quintiles*								
1 – Most deprived	188,963	22.8	13,591	27.8	47,157	22.8	3426	28.0
2	176,740	21.3	11,439	23.4	44,465	21.5	2796	22.9
3	153,201	18.5	9026	18.5	38,444	18.6	2361	19.3
4	155,458	18.8	8096	16.6	38,582	18.6	1995	16.3
5 – Least deprived	153,635	18.6	6736	13.8	38,338	18.5	1657	13.5
*Household size*								
1	184,491	22.3	12,150	24.9	46,081	22.3	3002	24.5
2	199,164	24.1	13,649	27.9	49,945	24.1	3457	28.3
3–5	404,298	48.8	21,200	43.4	100,990	48.8	5282	43.2
6–10	32,318	3.9	1604	3.3	8053	3.9	421	3.4
11+	7726	0.9	285	0.6	1917	0.9	73	0.6
*Urban–rural classification*								
Large urban areas	287,174	34.7	17,378	35.5	71,493	34.5	4404	36.0
Other urban areas	339,401	41.0	20,246	41.4	85,276	41.2	5013	41.0
Accessible small towns	73,951	8.9	4050	8.3	18,500	8.9	998	8.2
Remote small towns	29,331	3.5	1717	3.5	7267	3.5	456	3.7
Accessible rural	71,104	8.6	3589	7.3	17,728	8.6	913	7.5
Remote rural	27,036	3.3	1908	3.9	6722	3.2	451	3.7
*Variant period*								
Wild (up to 10 January 2021)	90,548	10.9	7777	15.9	22,642	10.9	1954	16.0
Alpha (11 January 2021–09 May 2021)	42,458	5.1	3697	7.6	10,599	5.1	944	7.7
Delta (24 May 2021–28 November 2021)	258,298	31.2	15,844	32.4	64,290	31.1	3898	31.9
Omicron (20 December 2021 onwards)	342,241	41.3	17,580	36.0	85,982	41.5	4467	36.5
No dominant variant or unknown	94,452	11.4	3990	8.2	23,473	11.3	972	7.9
*Vaccination doses (up to 14 days before positive test/outcome)*								
0	235,822	28.5	15,207	31.1	58,838	28.4	3773	30.8
1	63,472	7.7	2749	5.6	15,816	7.6	684	5.6
2	296,421	35.8	16,487	33.7	74,247	35.9	4104	33.5
3+	232,282	28.1	14,445	29.5	58,085	28.1	3674	30.0
*Shielding*								
Shielding	23,301	2.8	3764	7.7	5768	2.8	956	7.8
Not shielding	804,696	97.2	45,124	92.3	201,218	97.2	11,279	92.2
*Immunosuppressed*								
Immunosuppressed	24,251	2.9	3250	6.6	5987	2.9	847	6.9
Not immunosuppressed	803,746	97.1	45,638	93.4	200,999	97.1	11,388	93.1
*Care home resident*								
Care home resident	5616	0.7	226	0.5	1369	0.7	59	0.5
Not care home resident	822,381	99.3	48,662	99.5	205,617	99.3	12,176	99.5
*BMI (kg/m^2^)*								
Underweight (BMI < 18.5)	13,020	1.6	876	1.8	3221	1.6	203	1.7
Normal weight (BMI 18.5–24.9)	215,963	26.1	9784	20.0	54,239	26.2	2466	20.2
Overweight (BMI 25–29.9)	308,896	37.3	15,726	32.2	76,924	37.2	3819	31.2
Obese (BMI > 29.9)	290,118	35.0	22,502	46.0	72,602	35.1	5747	47.0
*Comorbidities*								
0	547,007	66.1	22,467	46.0	136,303	65.9	5623	46.0
1	198,465	24.0	15,510	31.7	50,157	24.2	3812	31.2
2	54,242	6.6	6639	13.6	13,565	6.6	1655	13.5
3+	28,283	3.4	4272	8.7	6961	3.4	1145	9.4
*Severity of acute infection (positive cases)*								
Hospitalised within 28 days	31,166	3.8	5127	10.5	7778	3.8	1270	10.4
Not hospitalised within 28	796,831	96.2	43,761	89.5	199,208	96.2	10,965	89.6

The table presents the number and percentage of individuals in the training and holdout datasets, classified as having long COVID or not according to the outcome measure.

### Model results

**Figure 3.**
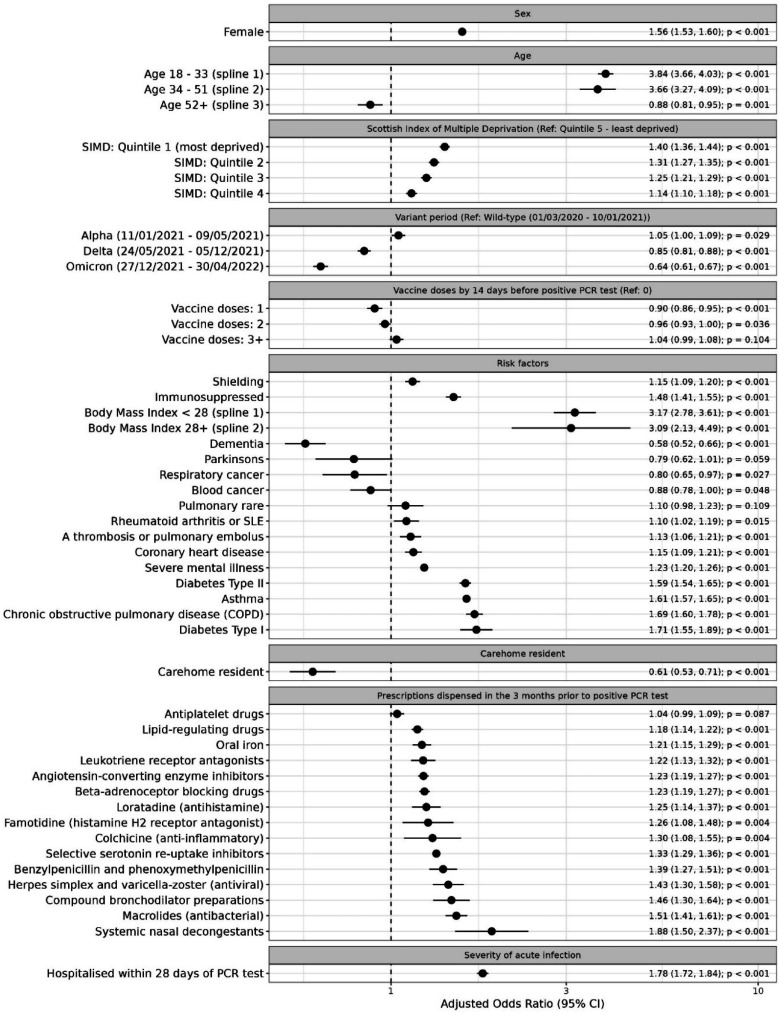
Adjusted odds ratios for predictors of long COVID. The plot illustrates the adjusted odds ratios and 95% confidence intervals for all predictors of long COVID included in the main multivariable logistic regression model. The model was trained on the training dataset (*n* = 882,782) using multivariable logistic regression with 10-fold cross-validation. SIMD quintiles relate to quintiles of the Scottish Index of Multiple Deprivation.

**Figure 4.**
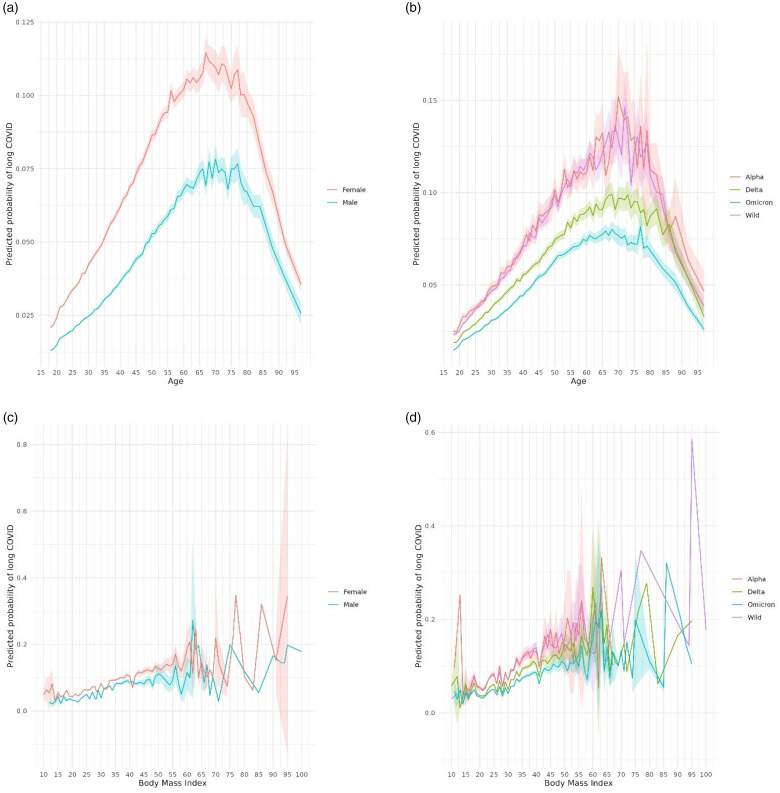
Predicted probability of long COVID by age, sex, BMI and variant period. (a) Predicted probabilities of long COVID by age and sex. (b) Predicted probabilities of long COVID by age and dominant SARS-CoV-2 variant in circulation when an individual received a positive RT-PCR test. (c) Predicted probabilities of long COVID by BMI and sex. (d) Predicted probabilities of long COVID by BMI and dominant SARS-CoV-2 variant in circulation when an individual received a positive RT-PCR test. Predicted probabilities were estimated by applying the main multivariable regression model to the training dataset (N = 876,885). Shading indicates 95% confidence intervals.

Figure 3 presents adjusted odds ratios (aORs) for each predictor, estimated using the multivariable logistic regression model with 10-fold cross validation (Equation S1). Increasing BMI and increasing age (up to 65–70 years) were associated with increased risk of long COVID. Female sex, severe acute COVID-19 infection, deprivation, immunosuppression, and being advised to shield were also associated with increased risk of long COVID. Figure 4 presents predicted probabilities by age, sex, BMI and variant period.

Eight of the 13 comorbidities investigated were associated with increased risk of long COVID (Type I and Type II diabetes, asthma, chronic obstructive pulmonary disease, severe mental illness, coronary heart disease, a thrombosis or pulmonary embolus, and rheumatoid arthritis or systemic lupus erythematosus. Dementia, respiratory cancer and blood cancer were associated with reduced risk of long COVID. All prescriptions included in the model were associated with an increased risk of long COVID, with the exception of antiplatelet drugs, for which there was no significant association.

Predictors associated with a lower risk of long COVID included testing positive while Omicron or Delta were dominant SARS-CoV-2 variants (relative to testing positive when the wild-type variant was dominant), having received one or two doses of COVID-19 vaccination (relative to being unvaccinated) and care home residency.

### Model evaluation in the holdout dataset

In the holdout dataset, the model achieved a c-statistic of 0.71 (95% CI: 0.71–0.72), area under the precision-recall curve of 0.13 (95% CI: 0.13–0.14) and a calibration slope of 1.01 (95% CI: 0.99–1.03) with the intercept at 0.01. Calibration plots (Figures S2–S3) indicated agreement between observed and predicted probabilities of developing long COVID, with some under-estimation at higher vigintiles of predicted probabilities.

At a discrimination threshold of 0.056 (observed prevalence of long COVID), sensitivity and specificity were 0.65 (95% CI: 0.64–0.66) and 0.67 (95% CI: 0.66–0.67), respectively. The model achieved an accuracy of 0.67 (95% CI: 0.66–0.67), PPV of 0.10 (95% CI: 0.10–0.11), NPV of 0.97 (95% CI: 0.97–0.97), F1 score of 0.18 (95% CI: 0.18–0.18), Matthew’s correlation coefficient of 0.15 (95% CI: 0.15–0.16) and Brier score of 0.33 (95% CI: 0.33–0.34) (Table S8).

### Internal validation

Across most metrics, model performance in the holdout dataset was comparable to that in the training dataset, with marginally higher F1 Score and Matthew’s correlation coefficient (Table S8) suggesting minimal overfitting. Subgroup analyses demonstrated consistency in model calibration across age and variant periods (Figures S4–S5).

### Results of sensitivity analyses

Results from the sensitivity analyses are presented below, with further detail provided in the Supplementary Material (pp.S20–S30).

#### Incorporating data from individuals with positive LFTs

Including individuals with positive LFTs increased the cohort size to 1,458,018. Training the model on a randomly selected 80% of the cohort resulted in patterns of predictors that were consistent with the main analysis, with the exception that having received three doses of COVID-19 vaccination was associated with significantly (*p* < 0.05) reduced risk of long COVID, compared with no significant association in the main analysis. (Figure S6). Model performance in holdout data was generally consistent with that of the main model (Table S9).

#### Omitting individuals with incomplete follow-up

Omitting individuals with incomplete follow-up (due to death, reinfection or where the index date was fewer than six months before the end of the study period) retained 94.1% of the cohort (*n* = 825,184 in the training dataset, *n* = 206,357 in the holdout dataset). The model trained on these data was consistent with the main model, with the exception that having received three doses of COVID-19 vaccination was associated with significantly (*p* < 0.05) increased risk of long COVID compared with no significant association in the main analysis (Figure S7). Model performance, evaluated in the restricted holdout dataset, was marginally better than the main model, though specificity and accuracy were marginally worse (Table S10).

#### Variations of the main outcome measure

We trained two additional models on outcome measures that: (1) omitted to use the operational definition for identification of long COVID patients, and (2) omitted to use blood tests within the operational definition for identification. Observed prevalence of long COVID was lower according to these measures (1.4% and 2.2%, respectively, Table S11). The resultant models were consistent with the main model with respect to associations between long COVID and sociodemographic and some clinical predictors (asthma, coronary heart disease, severe mental illness). However, fewer clinical and prescribing predictors were identified as significantly associated with long COVID (Figure S8). In holdout data, the performance of the two additional models deviated somewhat from the main model (Table S12). Most notably, both models had lower PPV and higher NPV than the main model, indicating a tendency to produce a higher rate of false positives and a lower rate of false negatives.

#### Models derived using machine learning

The XGBoost model exhibited good consistency with the main analysis in terms of the most important predictors of long COVID identified (Figure S9). When evaluated in holdout data, both machine learning approaches correctly identified more negative cases and fewer positive cases than the main model, resulting in higher accuracy (Table S13).

#### Model training and testing using a geographic split

The model trained on 12 of Scotland’s 14 geographic regions closely resembled the main model (Figure S10). In each holdout region, model performance was consistent with the main analysis in terms of the c-statistic and calibration slope, but generally worse in other metrics (Table S14).

### Discussion

In a cohort of 1.1 million adult residents in Scotland, we analysed coded and free-text data recorded in EHRs to derive and internally validate a long COVID risk prediction model. Whereas existing models rely on survey data,^6,7,11,12^ which may be prone to bias, or use EHR data^8–10^ that likely under-identifies long COVID cases, we combined clinical codes, free-text data and a comprehensive operational definition to improve case identification, in an effort to reduce under-reporting, and enhance prediction accuracy.

In-keeping with other studies, we identified several predictors of increased risk of long COVID, including: increasing age,^6–10^ increasing BMI,^6^ severe COVID-19 infection,^5,8,11^ female sex,^6,9,11,12^ increasing deprivation^6^ and several comorbidities^8,9,11^ and prescriptions. Infection with COVID-19 during the Delta and Omicron periods^11,23^ predicted reduced risk of long COVID, relative to the wild-type (though higher incidence of COVID-19 during these periods resulted in more long COVID cases in absolute terms). Vaccination^9,10^ also predicted reduced risk. While these results predict who is at risk of developing long COVID, the methods we used do not allow for a causal interpretation of our results.

This study has several strengths. Using data from the EAVE II platform enabled analysis of EHRs for a large, nationally representative cohort using an extensive range of clinically relevant predictors. Our main results were robust to variations in inclusion criteria, cohort subsets, modelling approaches and training and holdout splits. Involvement of PPI and clinicians enhanced the study design and interpretation. Our use of a multi-faceted long COVID identification method, which did not require explicit coding of long COVID in EHRs, lays a foundation for analyses of other poorly coded conditions.

Our study also has limitations. Compared to the models we trained using more conservative versions of the outcome measure, our main model identified more comorbidities and dispensed prescriptions as being significant predictors of long COVID. It is possible that, by identifying more long COVID cases for model training, the main outcome measure enhanced our ability to detect associations between long COVID and less prevalent predictors (compared with the more conservative variations of the outcome measure). However, we cannot rule out the possibility that the main outcome measure was biased towards misclassifying individuals with other health conditions as having long COVID.

Systematic biases in EHR data may also have influenced our results. For example, the positive association between long COVID and having received three or more COVID-19 vaccinations, which emerged during sensitivity analysis, likely reflects a confounding effect. This effect may have arisen because vulnerable populations, who were prioritised during vaccine roll-outs, typically exhibited higher rates of vaccine uptake.^24^ Moreover, the negative associations identified between long COVID and older adults, care home residents and dementia patients may reflect under-recording of indicators used to identify long COVID among these groups. More generally, reliance on EHR data excluded the experiences of individuals who had not interacted with the healthcare system, or whose interactions were not accurately recorded. Inconsistent recording of ethnicity and smoking status^25^ precluded investigation of these features. With the withdrawal of mass testing for COVID-19, it will not be possible to use the main outcome measure from this study to identify long COVID cases going forward.

We found that using machine learning techniques achieved higher accuracy, but more conservative prediction of positive cases, compared with the main logistic regression model. This highlights the potential value of these methods in situations where accuracy is prioritised over sensitivity. However, these methods offer less transparency than logistic regression in terms of the associations between predictors and outcomes.

The main model demonstrated reasonable discriminative ability and precision in holdout data; however, the degree of certainty offered by the model is not sufficient for use in clinical practice, given the risk of adverse outcomes from misclassification. Several factors likely contributed to the model’s modest performance. First, the relatively low observed prevalence of long COVID (5.6%) likely limited the model’s ability to accurately predict positive cases. Second, identifying long COVID cases from EHRs is inherently challenging, due to the lack of a universally accepted clinical definition and ongoing clinical uncertainty surrounding the condition.^4,5^ This uncertainty, coupled with under-utilisation of diagnostic codes,^13–15^ further complicates case identification. Additionally, unmeasured confounders – such as unrecorded symptoms – may have influenced model accuracy. Despite these challenges, our findings on risk factors for long COVID provide valuable insights for policymakers and public health tasked with developing preventive public health strategies or allocating and targeting resources to support long COVID patients. Our results could also aid researchers in identifying participants for inclusion in trials investigating preventive strategies or treatments for long COVID.

In conclusion, this study developed and internally validated a long COVID risk prediction model using EHR data and a novel case identification approach.

## Supplemental Material

sj-pdf-1-jrs-10.1177_01410768241297833 - Supplemental material for Deriving and validating a risk prediction model for long COVID: a population-based, retrospective cohort study in Scotland Supplemental material, sj-pdf-1-jrs-10.1177_01410768241297833 for Deriving and validating a risk prediction model for long COVID: a population-based, retrospective cohort study in Scotland by Karen Jeffrey, Vicky Hammersley, Rishma Maini, Anna Crawford, Lana Woolford, Ashleigh Batchelor, David Weatherill, Chris White, Tristan Millington, Robin Kerr, Siddharth Basetti, Calum Macdonald, Jennifer K Quint, Steven Kerr, Syed Ahmar Shah, Amanj Kurdi, Colin R Simpson, Srinivasa Vittal Katikireddi, Igor Rudan, Chris Robertson, Lewis Ritchie, Aziz Sheikh and Luke Daines in Journal of the Royal Society of Medicine

## Declarations

### Guarantor

KJ, LD and AS.

### Data sharing statement

All code, metadata and documentation for this project is publicly available at https://github.com/EAVE-II/Long-COVID. Most of the data used in this study are highly sensitive and will not be made available publicly.

### Provenance

Not commissioned; peer reviewed by Sun Jae Park and Julie Morris.
