# Supplementary material for: Deriving and validating a risk prediction model for long COVID: a population-based, retrospective cohort study in Scotland
Source: J R Soc Med. 2024 Nov 18;117(12):402–14. doi: 10.1177/01410768241297833 (PMC11574934; doi:10.1177/01410768241297833)
Supplement: sj-pdf-1-jrs-10.1177_01410768241297833 - Supplemental material for Deriving and validating a risk prediction model for long COVID: a population-based, retrospective cohort study in Scotland [file sj-pdf-1-jrs-10.1177_01410768241297833.pdf]

Deriving and validating a risk prediction model for long COVID: population-based, retrospective cohort study in Scotland

Contents

Reporting guidelines 2
Linked datasets 3
Outcome measure 4
Predictor selection 10
Patient and public involvement with this study 13
Sensitivity analyses....................20

Tables

Table S1: Transparent Reporting of Multivariable Prediction Models (TRIPOD) reporting checklist 2
Table S2: Patient comorbidities in training and holdout datasets, stratified by long COVID classification 6
Table S3: Dispensed prescriptions in training and holdout datasets, stratified by long COVID classification 7
Table S4: Dispensed prescriptions - British National Foundry (BNF) Sub-paragraph and Chemical Substance codes 8
Table S5: Results of predictor selection 11
Table S6: GRIPP2 reporting checklist (short form) 13
Table S7: Results of PPI involvement 13
Table S8: Evaluation metrics (95% confidence interval) for the multivariable logistic regression model in the training and holdout datasets 18
Table S9: Evaluation metrics (95% confidence interval) for the multivariable logistic regression model tested in holdout data, trained and tested on datasets containing (i) all individuals with a positive RT-PCR test result, and (ii) all individuals with a positive RT-PCR or LFT result 21
Table S10: Evaluation metrics (95% confidence interval) for the multivariable logistic regression model trained and tested on datasets containing individuals with complete follow up 23
Table S11: Variations of the long COVID outcome measure....................24
Table S12: Evaluation metrics (95% confidence interval) for the multivariable logistic regression model in the holdout dataset (n = 219,221), for variations on the outcome measure 26
Table S13: Evaluation metrics for models trained using multivariable logistic regression (main model), XGBoost, and a Naïve Bayes Classifier 28
Table S14: Evaluation metrics for the main multivariable logistic regression model compared and models trained and tested using a geographic split 30

Figures

Figure S1: Least absolute shrinkage and selection operator (LASSO) regression with resampling 12
Figure S2: Observed and predicted probabilities of long COVID at each vigintile of predicted probabilities in the training and holdout dataests 17
Figure S3: Smooth calibration plot 17
Figure S4: Observed and predicted probabilities at each vigintile of predicted probabilities, by age 18
Figure S5: Observed and predicted probabilities at each vigintile of predicted probabilities, by variant....................19
Figure S6: Adjusted odds ratios for predictors of long COVID estimated for all individuals with a positive RT-PCR or LFT result. 20
Figure S7: Adjusted odds ratios for predictors of long COVID for individuals with complete follow up 22
Figure S8: Adjusted odds ratios for predictors of long COVID estimated using alternative outcome measures 25
Figure S9: Feature importance scores estimated using a Gradient Boosted Decision Tree 27
Figure S10: Adjusted odds ratios for predictors of long COVID, trained in 12 of Scotland's 14 health boards 29

Equations

Equation 1: Multivariable logistic regression model specification (main model) 16

Reporting guidelines

The manuscript was guided by the Transparent Reporting of Multivariable Prediction Models (TRIPOD) reporting checklist (Table S1).

Table S1: Transparent Reporting of Multivariable Prediction Models (TRIPOD) reporting checklist

Section/Topic	Item		Checklist Item	Page
**Title and abstract**				
Title	1	D;V	Identify the study as developing and/or validating a multivariable prediction model, the target population, and the outcome to be predicted.	1
Abstract	2	D;V	Provide a summary of objectives, study design, setting, participants, sample size, predictors, outcome, statistical analysis, results, and conclusions.	2
**Introduction**				
Background and objectives	3a	D;V	Explain the medical context (including whether diagnostic or prognostic) and rationale for developing or validating the multivariable prediction model, including references to existing models.	4
	3b	D;V	Specify the objectives, including whether the study describes the development or validation of the model or both.	4
**Methods**				
Source of data	4a	D;V	Describe the study design or source of data (e.g., randomized trial, cohort, or registry data), separately for the development and validation data sets, if applicable.	4-5
	4b	D;V	Specify the key study dates, including start of accrual; end of accrual; and, if applicable, end of follow-up.	5
Participants	5a	D;V	Specify key elements of the study setting (e.g., primary care, secondary care, general population) including number and location of centres.	5
	5b	D;V	Describe eligibility criteria for participants.	5
	5c	D;V	Give details of treatments received, if relevant.	N/A
Outcome	6a	D;V	Clearly define the outcome that is predicted by the prediction model, including how and when assessed.	5
	6b	D;V	Report any actions to blind assessment of the outcome to be predicted.	N/A
Predictors	7a	D;V	Clearly define all predictors used in developing or validating the multivariable prediction model, including how and when they were measured.	6-7, Table S2-S3
	7b	D;V	Report any actions to blind assessment of predictors for the outcome and other predictors.	N/A
Sample size	8	D;V	Explain how the study size was arrived at.	5
Missing data	9	D;V	Describe how missing data were handled (e.g., complete-case analysis, single imputation, multiple imputation) with details of any imputation method.	7
Statistical analysis methods	10a	D	Describe how predictors were handled in the analyses.	7
	10b	D	Specify type of model, all model-building procedures (including any predictor selection), and method for internal validation.	7-8, Table S5, Figure S1
	10c	V	For validation, describe how the predictions were calculated.	8
	10d	D;V	Specify all measures used to assess model performance and, if relevant, to compare multiple models.	8
	10e	V	Describe any model updating (e.g., recalibration) arising from the validation, if done.	N/A
Risk groups	11	D;V	Provide details on how risk groups were created, if done.	N/A
Development vs. validation	12	V	For validation, identify any differences from the development data in setting, eligibility criteria, outcome, and predictors.	Table 1
**Results**				
Participants	13a	D;V	Describe the flow of participants through the study, including the number of participants with and without the outcome and, if applicable, a summary of the follow-up time. A diagram may be helpful.	8, Figure 1
	13b	D;V	Describe the characteristics of the participants (basic demographics, clinical features, available predictors), including the number of participants with missing data for predictors and outcome.	8, Table 1, Table S2-3
	13c	V	For validation, show a comparison with the development data of the distribution of important variables (demographics, predictors and outcome).	8, Table 1, Table S2-3
Model development	14a	D	Specify the number of participants and outcome events in each analysis.	8, Fig 1
	14b	D	If done, report the unadjusted association between each candidate predictor and outcome.	N/A

**Model specification**	**15a**	**D**	**Present the full prediction model to allow predictions for individuals (i.e., all regression coefficients, and model intercept or baseline survival at a given time point).**	**Equati on S1**
	**15b**	**D**	**Explain how to the use the prediction model.**	**9**
**Model performance**	**16**	**D;V**	**Report performance measures (with CIs) for the prediction model.**	**9**
**Model-updating**	**17**	**V**	**If done, report the results from any model updating (i.e., model specification, model performance).**	**N/A**
**Discussion**				
**Limitations**	**18**	**D;V**	**Discuss any limitations of the study (such as nonrepresentative sample, few events per predictor, missing data).**	**11**
**Interpretation**	**19a**	**V**	**For validation, discuss the results with reference to performance in the development data, and any other validation data.**	**9, Table S9-10, Figure S3-7**
	**19b**	**D;V**	**Give an overall interpretation of the results, considering objectives, limitations, results from similar studies, and other relevant evidence.**	**10-11**
**Implications**	**20**	**D;V**	**Discuss the potential clinical use of the model and implications for future research.**	**11-13**
**Other information**				
**Supplementary information**	**21**	**D;V**	**Provide information about the availability of supplementary resources, such as study protocol, Web calculator, and data sets.**	**through out**
**Funding**	**22**	**D;V**	**Give the source of funding and the role of the funders for the present study.**	**Abstrac t, 14**

Linked datasets

Pseudonymised identifiers of National Health Service (NHS) Scotland's Community Healthcare Index (CHI) were used to link the following datasets:

- Primary care
  - Primary care health records and demographic data (GP data)

- Secondary care
  - In-patient data from Scottish Morbidity Record 01 (SMR-01)
  - Intensive care admissions from the Scottish Intensive Care Society Audit Group (SICSAG)

- Prescribing
  - Dispensed prescriptions from the Prescribing Information System (PIS)

- Testing and vaccinations
  - Reverse transcriptase polymerase chain reaction (RT-PCR) and lateral flow testing (LFT) data from the Electronic Communication or Surveillance in Scotland (ECOSS)
  - Whole Genome Sequencing (WGS) data from the Centre of Genomics (COG)
  - Records of vaccinations, shielding, and immunocompromised individuals extracted from Public Health Scotland's (PHS) Turas Vaccination Management Tool (TVMT)

- Deaths
  - Death registry data from the National Records of Scotland (NRS)

Outcome measure

We used four measures to identify patients with long COVID, following an existing approach.[1]

1. Long COVID clinical codes
We used diagnostic codes for ongoing symptomatic COVID-19 (^ESCT1348648 or A7955) and Post-COVID 19 syndrome (^ESCT1348645 or AyuJC). These codes were introduced in Scottish primary care on March 9, 2021, and were informed by National Institute for health and Care Excellence (NICE) led working definitions of long COVID.

2. Free text mentions of long COVID recorded in primary care
To identify long COVID patients using free text recorded in primary care, we began by identifying terms used by primary care practitioners to indicate long COVID in the free text field of EHRs. Using natural language processing (NLP), we identified the phrases (up to five words) that were most frequently recorded in the free text field of primary care records during consultations where a long COVID clinical code was also recorded. We manually reviewed the most frequently occurring phrases to identify phrases that were unambiguously indicative of long COVID. The phrases identified were: "long covid", "post covid", "ongoing covid", "post coronavirus", "ongoing coronavirus" (including variations containing capitalisation or non-alphanumeric characters). We used a binary variable to indicate whether each individual in the cohort had any of the long COVID phrases recorded in the free text field of their primary care records. We assessed the possible impact of negations (for instance, "not long COVID") by examining the proportion of long COVID phrases that appeared within six words before or after a negation term and found this to be low (1.06%).

3. Free text mentions of long COVID on sick notes
We created a binary variable to indicate whether each patient had any of the long COVID phrases recorded in the free text field of a sick note issued in primary care (also known as fitness to work certificates or "fit notes"). We found the share of patients with long COVID phrases that appeared within six words of a negation term to be negligible (<0.00%).

4. Operational definition of long COVID
To identify long COVID patients who had neither a long COVID clinical code nor free text indicating long COVID recorded in their EHRs, we applied an existing operational definition that identifies patients as having long COVID based on patterns of clinical interactions recorded in their EHRs. The operational definition was developed using EHRs from the same cohort of adults as were included in this study, extended to include individuals who had tested negative (as well as those who had tested positive) for SARS-CoV-2, during the same study period. Full details of how the operational definition was developed are published elsewhere,[1] and summarised below.

First, matched analysis was used to identify individual clinical interactions that were indicative of long COVID. Time-varying propensity score matching in month-long intervals was used to prepare a matched cohort that contained pairs of individuals with positive (exposed cases) and negative (controls) RT-PCR test results for SARS-CoV-2, matched on propensity to receive a positive RT-PCR test in a given month. Propensity scores were estimated using: splines in age (with three degrees of freedom); sex; deprivation quintile; six-fold urban-rural classification; local authority of residence; household size; number of COVID-19 vaccine doses received up to 14 days before the RT-PCR test date used for matching; and number of RT-PCR tests taken by the RT-PCR test date used for matching; presence or absence of each of 22 clinical risk factors identified as being predictive of severe COVID-19 outcomes;[2] splines in BMI (with three degrees of freedom); and binary indicators of individuals' status as immunosuppressed, recommended to shield, or having been hospitalised or admitted to an ICU in the 12 months before testing. Within the matched cohort, individual Poisson regression models were used to estimate adjusted rate ratios (aRR) for exposed cases, relative to control cases, in terms of a long list of clinical interactions that were considered to be potential indicators of long COVID (identified through a literature review and informed by the clinical expertise of the research team). The potential indicators included: 45 groups of clinical codes (each reflecting symptoms or diagnoses recorded in primary care records, for instance 18 codes relating to "dry cough", "chesty cough", "night cough" etc. were grouped as "cough"); 27 newly dispensed categories of prescriptions (whereby 'newly dispensed' refers to prescriptions that had not been dispensed in the 12 months prior to the test date

used for matching); and seven indicators of health service use (including counts of: GP visits, hospital admissions, outpatient attendances for respiratory conditions, A&E visits, out of hours encounters, intensive care unit (ICU) admissions, and NHS 24 telehealth interactions)). In each model, the dependent variable was counts of the potential indicator under investigation, as recorded in EHRs within 4-12 weeks and >12-26 weeks of the exposed case's test date in each matched pair. Models included an offset for days of follow-up and all predictors used in the propensity score estimation were included as covariates. The Quasi-Poisson variant of Poisson regression was used to adjust for the possibility of overdispersion. P-values were adjusted to reduce the false discovery rate, following Benjamini and Hochberg's approach. All clinical interactions that occurred at a significantly (adjusted-p < .05) higher rate among exposed individuals, relative to controls, were considered to be indicators of long COVID.

The potential indicators of long COVID were then classified into three categories: symptoms, investigations, and management strategies (Figure 2). This classification system was informed by the clinical expertise of the project's steering group.

According to the operational definition, any individual who had received a positive RT-PCR test, and who also had indicators from two or more of the three categories recorded in their EHRs during the 4-26 weeks following their RT-PCR test date, would be considered to be a long COVID patient.

Table S2: Patient comorbidities in training and holdout datasets, stratified by long COVID classification

	Training dataset				Holdout dataset			
	No long COVID		Long COVID		No long COVID		Long COVID	
	N	%	N	%	N	%	N	%
Total	827,997	94.4	48,888	5.6	206,986	94.4	12,235	5.6
Asthma	112,199	13.6	10,688	21.9	28,074	13.6	2,693	22.0
Atrial fibrillation	11,334	1.4	1,203	2.5	2,786	1.3	355	2.9
Chronic Kidney disease (level 3+)	16,531	2.0	1,868	3.8	4,072	2.0	498	4.1
Chronic obstructive pulmonary disease (COPD)	13,071	1.6	2,739	5.6	3,238	1.6	678	5.5
Coronary heart disease	21,302	2.6	3,029	6.2	5,366	2.6	775	6.3
Dementia	7,187	0.9	301	0.6	1,865	0.9	88	0.7
Diabetes Type I	3,870	0.5	467	1.0	986	0.5	100	0.8
Diabetes Type II	35,529	4.3	5,756	11.8	8,865	4.3	1,414	11.6
Epilepsy	10,313	1.2	734	1.5	2,622	1.3	200	1.6
Fracture	32,047	3.9	2,176	4.5	8,163	3.9	570	4.7
Haematological cancer	2,862	0.3	313	0.6	673	0.3	88	0.7
Heart failure	5,068	0.6	669	1.4	1,186	0.6	196	1.6
Neurological disorder	2,844	0.3	229	0.5	688	0.3	55	0.4
Parkinsonâ€™s disease	1,166	0.1	75	0.2	285	0.1	25	0.2
Peripheral vascular disease	4,144	0.5	544	1.1	951	0.5	144	1.2
Pulmonary hypertension	901	0.1	115	0.2	220	0.1	35	0.3
Rare pulmonary disease	2,707	0.3	416	0.9	655	0.3	104	0.9
Respiratory cancer	1,060	0.1	115	0.2	251	0.1	41	0.3
Rheumatoid arthritis or systemic lupus erythematous (SLE)	6,430	0.8	878	1.8	1,652	0.8	267	2.2
Severe mental illness	90,080	10.9	9,264	18.9	22,629	10.9	2,286	18.7
Stroke/Transient Ischaemic Attack (TIA)	13,378	1.6	1,498	3.1	3,272	1.6	388	3.2
Thrombosis or pulmonary embolus	9,594	1.2	1,095	2.2	2,392	1.2	264	2.2

The table presents the number and percentage of individuals in the training and holdout datasets with each comorbidity, classified as having long COVID or not according to our outcome measure. Percentages in the 'Total' row reflect the share of individuals classified as having long COVID or not as a share of the dataset total. Neurological disorder includes motor neurone disease, multiple sclerosis, myasthenia gravis and Huntington's chorea. Rare pulmonary disease includes cystic fibrosis, bronchiectasis or alveolitis. Severe mental illness includes bipolar affective disorder, psychosis, schizophrenia or schizoaffective disorder, and severe depression. The comorbidities selected for inclusion were informed by previous work.[2]

Table S3: Dispensed prescriptions in training and holdout datasets, stratified by long COVID classification

	Training dataset				Holdout dataset			
	No long COVID		Long COVID		No long COVID		Long COVID	
	N	%	N	%	N	%	N	%
Total	827,997	94.4	48,888	5.6	206,986	94.4	12,235	5.6
Alpha-adrenoceptor blocking drugs	5,033	0.6	653	1.3	1,237	0.6	188	1.5
Angiotensin-converting enzyme inhibitors	49,990	6.0	6,218	12.7	12,431	6.0	1,586	13.0
Antiplatelet drugs	36,936	4.5	4,711	9.6	9,343	4.5	1,238	10.1
Benzylpenicillin and phenoxymethylpenicillin	8,045	1.0	581	1.2	2,079	1.0	131	1.1
Beta-adrenoceptor blocking drugs	56,467	6.8	6,346	13.0	14,156	6.8	1,656	13.5
Colchicine (anti-inflammatory)	1,367	0.2	142	0.3	366	0.2	40	0.3
Compound bronchodilator preparations	1,939	0.2	436	0.9	470	0.2	98	0.8
Corticosteroid replacement therapy	588	0.1	56	0.1	186	0.1	17	0.1
Direct oral anticoagulants	10,974	1.3	1,236	2.5	2,725	1.3	326	2.7
Famotidine (histamine H2 receptor antagonist)	1,392	0.2	187	0.4	355	0.2	54	0.4
Herpes simplex and varicella-zoster (antiviral)	4,788	0.6	490	1.0	1,201	0.6	125	1.0
Leukotriene receptor antagonists	5,410	0.7	954	2.0	1,393	0.7	235	1.9
Lipid-regulating drugs	70,265	8.5	9,113	18.6	17,566	8.5	2,318	18.9
Loratadine (antihistamine)	5,035	0.6	592	1.2	1,263	0.6	150	1.2
Macrolides (antibacterial)	8,095	1.0	1,127	2.3	2,113	1.0	273	2.2
Oral iron	12,946	1.6	1,492	3.1	3,248	1.6	382	3.1
Parenteral anticoagulants	989	0.1	91	0.2	257	0.1	17	0.1
Ranitidine hydrochloride	277	0.0	40	0.1	47	0.0	6	0.0
Selective serotonin re-uptake inhibitors	85,095	10.3	8,725	17.8	21,308	10.3	2,229	18.2
Systemic nasal decongestants	696	0.1	88	0.2	180	0.1	20	0.2
Ursodeoxycholic acid	568	0.1	73	0.1	151	0.1	26	0.2
Warfarin sodium	2,990	0.4	323	0.7	777	0.4	93	0.8

The table presents the number and percentage of individuals in the training and holdout datasets dispensed each prescription during the three months before receiving their first positive RT-PCR test, classified as having long COVID or not according to our outcome measure. Percentages in the ‘Total’ row reflect the share of individuals classified as having long COVID or not as a share of the dataset total.

Table S4: Dispensed prescriptions - British National Foundry (BNF) Sub-paragraph and Chemical Substance codes

BNF Chapter	BNF Section	BNF Subparagraph	BNF Chemical Substances	Group used in analysis
Cardiovascular System	Beta-adrenoceptor blocking drugs	Beta-adrenoceptor blocking drugs (204000)	All substances in subparagraph	Beta-adrenoceptor blocking drugs
	Hypertension and heart failure	Alpha-adrenoceptor blocking drugs (205040)	All substances in subparagraph	Alpha-adrenoceptor blocking drugs
		Angiotensin-converting enzyme inhibitors (205051)	All substances in subparagraph	Angiotensin-converting enzyme inhibitors
	Anticoagulants and protamine	Parenteral anticoagulants (208010)	All substances in subparagraph	Parenteral anticoagulants
		Oral anticoagulants (208020)	Apixaban (0208020Z0)	Direct oral anticoagulants
			Dabigatran etexilate (0208020X0)	
			Edoxaban (0208020AA)	
			Rivaroxaban (0208020Y0)	
			Warfarin sodium (0208020V0)	Warfarin sodium
	Antiplatelet drugs	Antiplatelet drugs (209000)	All substances in subparagraph	Antiplatelet drugs
	Lipid-regulating drugs	Lipid-regulating drugs (212000)	All substances in subparagraph	Lipid-regulating drugs
Respiratory System	Bronchodilators	Selective beta(2)-agonists (301011)	All substances in subparagraph	Selective beta(2)-agonists*
		Compound bronchodilator preparations (301040)	All substances in subparagraph	Compound bronchodilator preparations
	Corticosteroids (respiratory)	Corticosteroids (respiratory) (302000)	All substances in subparagraph	Inhaled corticosteroids*
	Cromoglycate, leukotriene and phosphodesterase type-4 inhib	Leukotriene receptor antagonists (303020)	All substances in subparagraph	Leukotriene receptor antagonists
	Antihistamines, hyposensitisation and allergic emergencies	Antihistamines (304010)	Loratadine (0304010D0)	Loratadine
	Cough preparations	Cough suppressants (309010)	All substances in subparagraph	Cough suppressants*
		Expectorant and demulcent cough preparations (309020)	All substances in subparagraph	Expectorant and demulcent cough preparations*
	Systemic nasal decongestants	Systemic nasal decongestants (310000)	All substances in subparagraph	Systemic nasal decongestants
Infections	Antibacterial drugs	Benzylpenicillin and phenoxymethylpenicillin (501011)	All substances in subparagraph	Benzylpenicillin and phenoxymethylpenicillin
		Tetracyclines (501030)	All substances in subparagraph	Tetracyclines*
		Macrolides (501050)	All substances in subparagraph	Macrolides
	Antiviral drugs	Herpes simplex and varicella-zoster (503021)	All substances in subparagraph	Herpes simplex and varicella-zoster
		Coronavirus (503060)	All substances in subparagraph	Antivirals to treat coronavirus*

BNF Chapter	BNF Section	BNF Subparagraph	BNF Chemical Substances	Group used in analysis
Endocrine System	Corticosteroids (endocrine)	Replacement therapy (603010)	All substances in subparagraph	Replacement therapy
	Drugs used in diabetes	Biguanides (601022)	Metformin hydrochloride (0601022B0)	Metformin hydrochloride
Nutrition and Blood	Anaemias and some other blood disorders	Oral iron (901011)	All substances in subparagraph	Oral iron
Central Nervous System	Antidepressant drugs	Selective serotonin re-uptake inhibitors (403030)	All substances in subparagraph	Selective serotonin re-uptake inhibitors
Gastro-Intestinal System	Drugs affecting intestinal secretions	Drugs affecting biliary composition and flow (109010)	Ursodeoxycholic acid (0109010U0)	Ursodeoxycholic acid
	Antisecretory drugs and mucosal protectants	H2-Receptor antagonists (103010)	Famotidine (0103010H0)	Famotidine
			Ranitidine hydrochloride (0103010T0)	Ranitidine hydrochloride
Musculoskeletal and Joint Diseases	Drugs used in rheumatic diseases and gout	Gout and cytotoxic induced hyperuricaemia (1001040)	Colchicine (1001040G0)	Colchicine

The table shows the British National Foundry Chemical Substances included in each of the 29 groups of prescriptions included in the analysis (indicated in the final column). The six groups marked with an asterisk were used to identify cases of long COVID for our outcome measure. All other groups of prescriptions were entered into the model as predictors.

Predictor selection
To identify a parsimonious set of predictors that maximised model fit, we ran backward stepwise selections to maximise (i) Akaike Information Criterion (AIC) and (ii) Bayesian Information Criterion (BIC) scores. We compared the fit of the resultant models to the full model using maximum likelihood ratio tests. AIC selection removed 12 predictors and produced a fit that was statistically indistinguishable from the fit of original model. BIC selection removed 26 predictors and resulted in significantly worse model fit ($p<.001$). We therefore used the subset of predictors retained during AIC selection.

To test the robustness of our predictor selection, we estimated least absolute shrinkage and selection operator (LASSO) models with resampling. To achieve this, we first identified the optimal λ (penalty term) using 10-fold cross-validation. We then estimated a LASSO model using a randomly selected 50% subset of the training data. We repeated this process 1,000 times and plotted the proportion of models each predictor was selected in (Figure S1). This allowed us to identify the predictors that were consistently selected across different resampled datasets. Reassuringly, all predictors that were deselected during AIC selection were selected in fewer than 95% of LASSO models. Six further predictors were selected in fewer than 95% of the LASSO models. Excluding these additional predictors led to no significant difference in c-statistic or area under the precision-recall curve achieved when modelling; we therefore omitted the additional six predictors from our model.

Table S5: Results of predictor selection

Predictors included in full model		Removed during AIC selection	Removed during BIC selection
Socio-demographic	Sex		
	Age		
	SIMD quintiles		
	Household size		
	Six-fold urban-rural classification		
Clinical	Variant period		
	Vaccine doses		
	Shielding		
	Immunosuppressed		
	Care home resident		*
	Body Mass index		
	Atrial fibrillation		*
	Asthma		
	Blood cancer		*
	Heart failure		*
	Coronary heart disease		
	Chronic kidney disease (level 3+)		*
	Chronic obstructive pulmonary disease (COPD)		
	Dementia		
	Diabetes Type I		
	Diabetes Type II		
	Epilepsy	*	*
	Fracture	*	*
	Neurological disorder	*	*
	Parkinson’s disease		*
	Pulmonary hypertension	*	*
	Rare pulmonary disease		*
	Peripheral vascular disease	*	*
	Rheumatoid arthritis or systemic lupus erythematous (SLE)		*
	Respiratory cancer		*
	Severe mental illness		
	Stroke/Transient ischaemic attach (TIA)	*	*
	Thrombosis or pulmonary embolus		*
	Severe acute COVID-19 infection		
Prescriptions	Lipid-regulating drugs		
	Angiotensin-converting enzyme inhibitors		
	Beta-adrenoceptor blocking drugs		
	Oral iron		
	Selective serotonin re-uptake inhibitors		
	Loratadine		
	Direct oral anticoagulants	*	*
	Colchicine		*
	Antiplatelet drugs		*
	Alpha-adrenoceptor blocking drugs	*	*
	Macrolides		
	Benzylpenicillin and phenoxymethylpenicillin		
	Leukotriene receptor antagonists		
	Herpes simplex and varicella-zoster		
	Replacement therapy	*	*
	Famotidine		*
	Ursodeoxycholic acid	*	*
	Systemic nasal decongestants		
	Warfarin sodium		*
	Parenteral anticoagulants	*	*
	Compound bronchodilator preparations		
	Ranitidine hydrochloride	*	*

The table identifies predictors removed during backward stepwise selection to optimise Akaike’s Information Criterion (AIC) and the Bayesian Information Criterion (BIC).

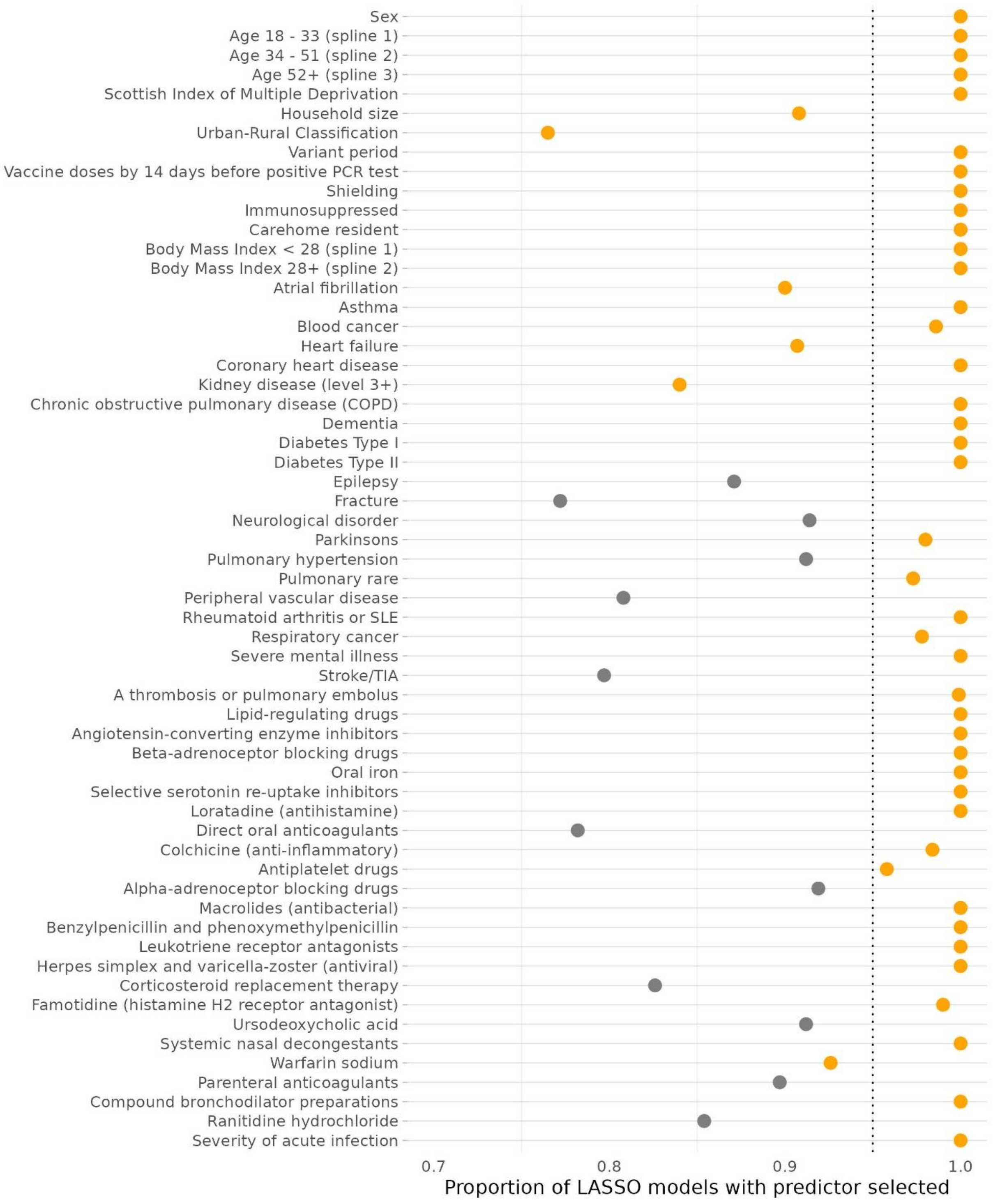

Figure S1: Least absolute shrinkage and selection operator (LASSO) regression with resampling
The figure shows the proportion of LASSO models that each predictor was selected in. Each model was derived using a randomly selected 50% subset of the training data. We repeated this process 1,000 times. Points to the right of the dotted line were selected in at least 95% of models. Grey points indicate predictors that were removed during backward selection to maximise Akaike's Information Criterion (AIC) scores.

Patient and public involvement with this study

Table S6: GRIPP2 reporting checklist (short form)

Section and topic	Item	Reported on page No
**1: Aim**	Report the aim of PPI in the study	S13
**2: Methods**	Provide a clear description of the methods used for PPI in the study	S13
**3: Study results**	Outcomes—Report the results of PPI in the study, including both positive and negative outcomes	S14
**4: Discussion and conclusions**	Outcomes—Comment on the extent to which PPI influenced the study overall. Describe positive and negative effects	S14
**5: Reflections/critical perspective**	Comment critically on the study, reflecting on the things that went well and those that did not, so others can learn from this experience	S14-15

PPI=patient and public involvement

Background to PPI involvement

The long Covid PPI team was established in October 2020, when Lay Co-Investigator, David Weatherill [DW] reviewed the initial bid. He was recruited based on his experience of working with health data and his co-leadership role on the EAVE II Public Advisory Group (PAG).[3] In July 2021, PPI co-ordinator, Lana Woolford [LW] recruited two additional partners, Ashleigh Batchelor [AB] and Chris White [CW] from the Long Covid Scotland Action Group. LW was succeeded by Anna Crawford [AC] from 1 March 2023.

PPI activities included commenting on the analysis protocol, steering the project, co-producing a PPI strategy, releasing a patient survey to inform analysis interpretation, reviewing plain English summaries of project outputs, assisting with public and policy documents released with study outputs, engaging with media and authoring a GRIPP2 appendix.

Aims of PPI involvement

The aims of patient and public involvement (PPI) in this study were to: (1) embed patient and public perspectives and information needs into project decision-making; (2) ensure that the experiences of people with Long Covid were incorporated into the study design; and (3) contribute to shared best practice in PPI.

Methods of PPI involvement

The PPI team has participated in 18 steering meetings and additional sessions to provide input and feedback into the project and steer decision making towards relevant priorities. This work was carried out remotely, either using video-conferencing software (Zoom, with minutes produced from each recording) or asynchronously via email. Public members of the PPI Team were paid for time and expertise shared in line with National Institute for Health and Care Research (NIHR) guidelines,[4] with appropriate paperwork issued to prevent compromise of any state financial support received.

Results of PPI involvement

PPI involvement over the entire duration of the study is documented in **Table S7**.

Table S7: Results of PPI involvement

Area of research cycle	Summary of deliverables
**Grant development**	Appoint Lay Co-Investigator and comment on grant application.
**Undertaking project**	Collaborate with Long Covid Scotland; co-produce PPI Strategy and Terms of Engagement; participate in induction and statistical methods training for project.
**Design**	Review analysis protocol; support design and release of survey gauging symptoms and impact of Long Covid on patients in Scotland; continue to question and comment on design development at Steering Group and PPI meetings.

**Analysis and interpretation**	Share results from Long Covid Scotland with analysts to inform interpretation; design and carry out consultation with people with Long Covid to select features for prediction model from patient perspective. Participate in steering group meetings and a workshop on selection of predictor variables.
**Dissemination**	Contribute to and review public-facing outputs to produce plain English resources and identify potential questions; collaborate with staff to provide written contributions for academic publications.
**Implementation**	Provide a steer on appropriate messaging and content to be released in policy briefing(s) and in any supporting media materials.
**Evaluation**	Evaluate PPI element of project in final stages; share this work by means of a PPI report.

Through engagement in the project's steering group meetings, the PPI team members provided insights that resulted in greater clarity of technical terminology used, deeper understanding of variations of long Covid presentations, approaches to capture the experience of positive COVID-19 cases in the absence of positive test results, and awareness of limitations in terms of the accuracy of information recorded in EHR. PPI members also prompted discussion and consideration of the impact of COVID-19 variants on individuals; patient awareness; presentation of survey results; and challenges of accessing medical records.

In addition to the Steering Group meetings, PPI contributors participated in a workshop to provide insights and reflections on the early results of the risk prediction model. Specifically, PPI members provided input on predictor selection and choice of discrimination thresholds. Observations involving the exclusion of ethnicity data, the use of sick notes in the definition of long Covid, the impact of certain medications on the data, queries on the socio-economic breakdown and exploring the relationship between variant types and vaccine protection were addressed. This input directly shaped the formulation and validation of the risk prediction model.

Discussion and conclusions from PPI involvement

Extensive input of PPI throughout the long Covid project has been instrumental in providing rich insights that have ensured the relevance of findings. PPI input was particularly beneficial in terms of ensuring project outputs were informed by the experiences of the types of individuals who stand to benefit most from a risk prediction model for COVID-19.

Reflections on PPI involvement

PPI contributors reported their involvement has been "valuable" and "meaningful" throughout the project. CW noted that the "team recognises the patient perspective" which "brings high value to the study". PPI members' insights have assisted in providing greater understanding of the analysis and findings. In particular, PPI members drew on their lived and real-world experiences of living with a long-term condition to shape the research. CW denoted the importance of PPI involvement in the steering group meetings as they "provide a space to ask questions and to comment from personal perspectives". Transparency was paramount and certain operations were re-considered to encourage accessibility such as, scheduling meetings at times to ensure inclusivity. CW emphasised the value of this "recognition" and felt "particularly proud" of the team dynamic and assuredness towards PPI contributions.

From a researcher's perspective, LD denoted the project has been "far richer" due to PPI involvement, which guided the study design and reporting. The team employed varying levels of expertise to solve problems, facilitate effectively and encourage active contribution. LD reported his 8 years of previous PPI experience endowed him with "familiarity" of public involvement which ensured accurate and realistic activities were supported by the team.

The work has been "worthwhile" and led to widespread public interest driven by media opportunities such as, the BBC presenting statistics from the project to represent the challenges of those living with long Covid. Operationally, multiple stakeholders were engaged through conversations at steering groups that enabled multi-disciplinary collaboration, relevant outputs and achievable objectives. As reported by VH, the timing of the first publication on the prevalence of Long Covid coincided with the Long Covid Inquiry Report and further impact was presented via the Royal Statistical Society "Florence Nightingale Award". Notably, CW emphasised his appreciation to the team for identifying the clinical and practice needs in the policy briefing to ensure the patient experience was accurately represented. Importantly, collaborating with significant media channels and organisations such as, the BBC provided far-reaching exposure to the significance of long Covid research.

Overall, VH noted deliverables were met with support from a “no-cost extension” and the study provided evidence of likely prevalence for those living with long Covid across Scotland. The methods used to extract information from GP patient data was quoted as being “novel and worked well”. This informed the relevance of the searches conducted. However, DW identified the lack of “ethnicity data” available that may have reduced the “usefulness” of the study on exploring the COVID-19 impact for those from an ethnic background.

An additional limitation was access to primary care data. This led to project changes with the organisation of a PPI workshop. This was “not anticipated” by the researchers and was “frustrating” for public contributors as “PPI meetings and input were frequently delayed”. Study progression was halted intermittently leading to impacts on analysis and results stages. Thus, LD noted the difficulty of “managing expectations” of the steering group in the first 12-18 months as well as new project leadership within the first 6 months that also resulted in postponements.

Nevertheless, statistical analysis training was requested by contributors and despite delays to the project, this was addressed. However, it was recommended by DW to be introduced at “an earlier stage” and the ability to access data more easily may be solved by making software such as, Adobe Acrobat, available to PPI team members. In the future, programmed timescales should be more “realistic and pressures should be applied if deadlines are missed”.

The above reflections were obtained from evaluations conducted by our Lay Co-Investigator, David Weatherill [DW], Long Covid PPI Group Representative, Chris White [CW], Research Lead, Dr Luke Daines [LD] and Project Manager, Dr Vicky Hammersley [VH], based on a framework created by Dr Lana Woolford [LW].

Equation 1: Multivariable logistic regression model specification (main model)

$$ln\left(\frac{p_{(long\ COVID)}}{1-p_{(long\ COVID)}}\right) =$$

- 4.41912
+ 0.45 (Sex: Female)
+ 1.35 (Age 18 - 33 (spline 1)) + 1.30 (Age 34 - 51 (spline 2)) - 0.13 (Age 52+ (spline 3))
+ 0.34 (SIMD quintile: 1) + 0.27 (SIMD quintile: 2) + 0.22 (SIMD quintile: 3) + 0.13 (SIMD quintile: 4)
+ 0.05 (Variant period: Alpha) – 0.17 (Variant period: Delta) - 0.44 (Variant period: Omicron) - 0.64 (Variant period: No dominant variant or Unknown)
- 0.10 (Vaccine doses: 1) - 0.04 (Vaccine doses: 2) + 0.03 (Vaccine doses: 3+)
+ 0.14 (Shielding)
+ 0.39 (Immunosuppressed)
- 0.49 (Care home resident)
+ 1.15 (BMI < 28 (spline 1)) + 1.13 (BMI 28+ (spline 2))
+ 0.47 (Asthma)
-0.13 (Haematological cancer)
+ 0.14 (Coronary heart disease)
+ 0.52 (Chronic obstructive pulmonary disease (COPD))
-0.54 (Dementia)
+ 0.54 (Diabetes Type I)
+ 0.47 (Diabetes Type II)
- 0.23 (Parkinsons)
+ 0.09 (Rare pulmonary disease)
+ 0.10 (Rheumatoid arthritis or systemic lupus erythematous (SLE))
- 0.23 (Respiratory cancer)
+ 0.21 (Severe mental illness)
+ 0.12 (Thrombosis or pulmonary embolus)
+ 0.20 (Angiotensin-converting enzyme inhibitors)
+ 0.04 (Antiplatelet drugs)
+ 0.33 (Benzylpenicillin and phenoxymethylpenicillin)
+ 0.21 (Beta-adrenoceptor blocking drugs)
+ 0.26 (Colchicine (anti-inflammatory))
+ 0.38 (Compound bronchodilator preparations)
+ 0.23 (Famotidine (histamine H2 receptor antagonist))
+ 0.36 (Herpes simplex and varicella-zoster (antiviral))
+ 0.20 (Leukotriene receptor antagonists)
+ 0.17 (Lipid-regulating drugs)
+ 0.22 (Loratadine (antihistamine))
+ 0.41 (Macrolides (antibacterial))
+ 0.19 (Oral iron)
+ 0.28 (Selective serotonin re-uptake inhibitors)
+ 0.63 (Systemic nasal decongestants)
+ 0.4 (Severe acute COVID19)

The equation specifies the main multivariable logistic regression model, estimated using the training dataset and fine-tuned using 10-fold cross validation. Reference categories were: Sex: Male; SIMD quintile: 5; Variant period: Wild-type (up to 10/01/2021); Vaccine doses: 0.

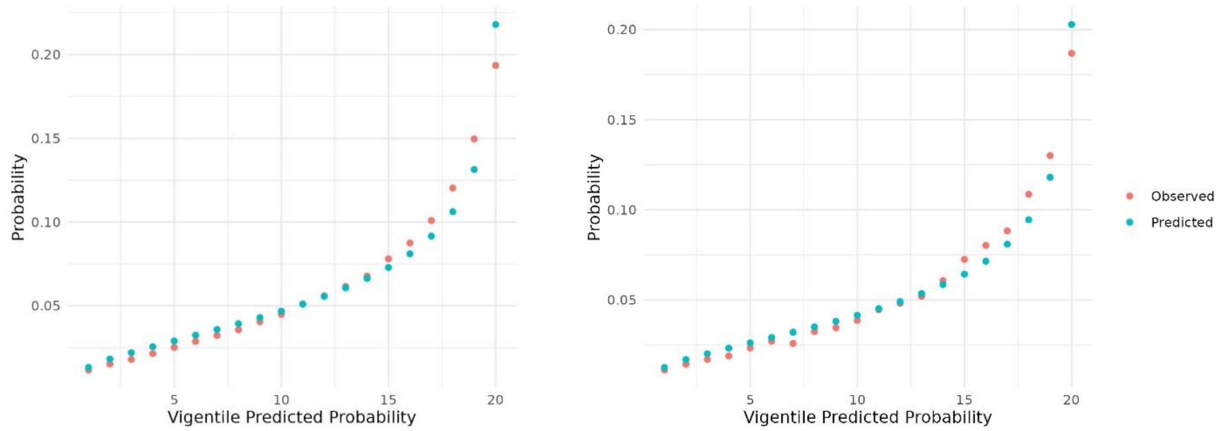

A. Training dataset **B. Holdout dataset**

Figure S2: Observed and predicted probabilities of long COVID at each vigintile of predicted probabilities in the training and holdout dataests

The plots illustrate the observed and predicted probabilities of long COVID at each vigintile of predicted probabilities. Panel A plots observed and predicted probabilities in the training dataset (n=876,885). Panel B plots observed and predicted probabilities in the holdout dataset (n=219,221).

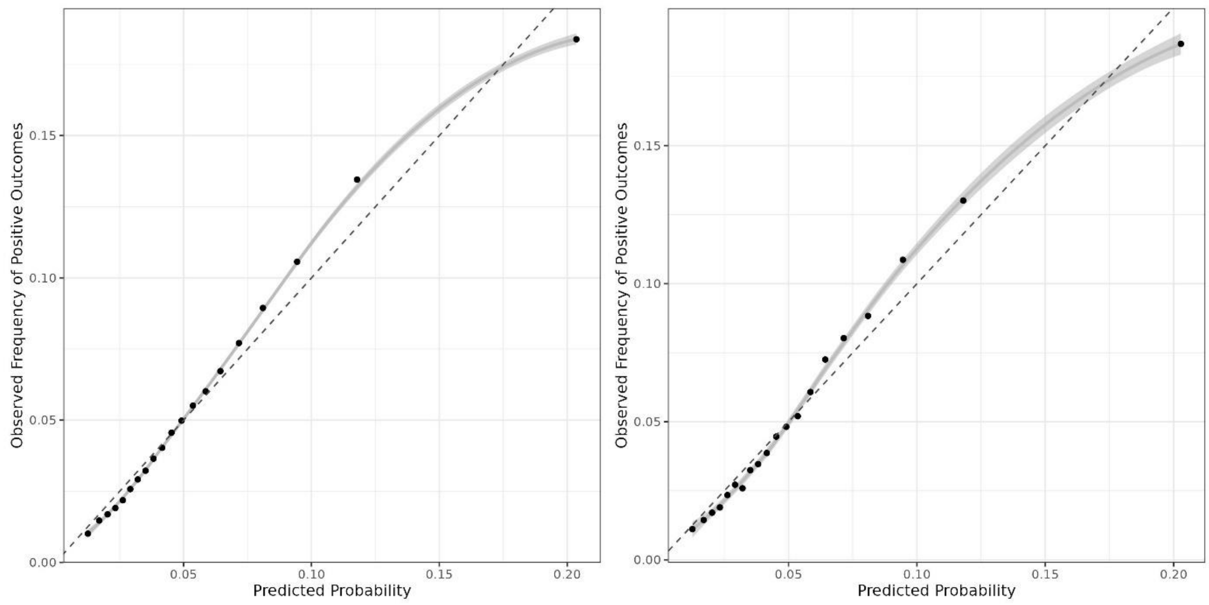

A. Calibration in the training dataset B. Calibration in the holdout dataset

Figure S3: Smooth calibration plot

The plots visualize the relationship between predicted probabilities (x-axis) and observed proportions (y-axis) of the binary outcome at each probability. Points represent observations in each predicted probability bin (vigintiles). The solid line indicates the calibration slope (Loess smoother). The shaded area represents the 95% confidence interval. The dashed line indicates perfect prediction. Panel A shows calibration in the training dataset (N = 876,885). Panel B shows calibration in the holdout dataset (N = 219,221).

Table S8: Evaluation metrics (95% confidence interval) for the multivariable logistic regression model in the training and holdout datasets

	Training data	Holdout data
N	876,885	219,221
C-statistic	0.713 (0.711-0.715)	0.714 (0.710-0.719)
AUC precision-recall	0.133 (0.131-0.135)	0.136 (0.133-0.139)
Calibration slope	1.000 (0.988-1.012)	1.010 (0.986-1.034)
Discrimination threshold (equal to prevalence of the dependent variable)	*0.056*	*0.056*
Sensitivity (Recall)	0.644 (0.640-0.648)	0.651 (0.643-0.660)
Specificity	0.667 (0.666-0.668)	0.668 (0.666-0.670)
Accuracy	0.666 (0.665-0.667)	0.667 (0.665-0.669)
Positive predicted value (PPV)	0.103 (0.101-0.104)	0.104 (0.102-0.106)
Negative predicted value (NPV)	0.969 (0.969-0.970)	0.970 (0.969-0.971)
F1 Score	0.177 (0.176-0.178)	0.179 (0.178-0.181)
Matthew's correlation coefficient	0.150 (0.149-0.151)	0.154 (0.152-0.156)
Brier score	0.334 (0.333-0.335)	0.333 (0.331-0.335)

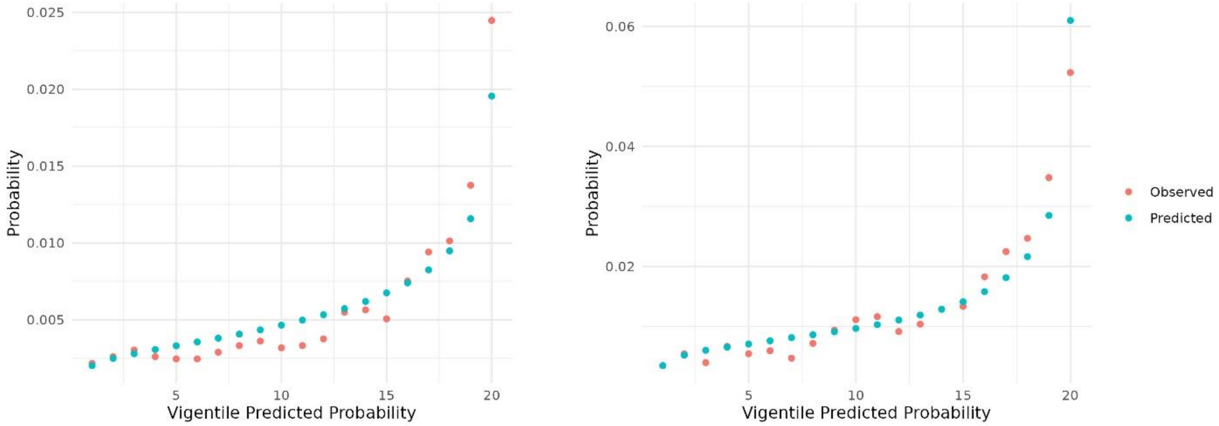

A. Aged under 50 B. Aged 50 and over

Figure S4: Observed and predicted probabilities at each vigintile of predicted probabilities, by age
The plots illustrate the observed and predicted probabilities of long COVID at each vigintile of predicted probability in our holdout dataset (N = 219,221), stratified by age. Panel A presents data for individuals under 50 years old. Panel B present data for individuals aged 50 and over.

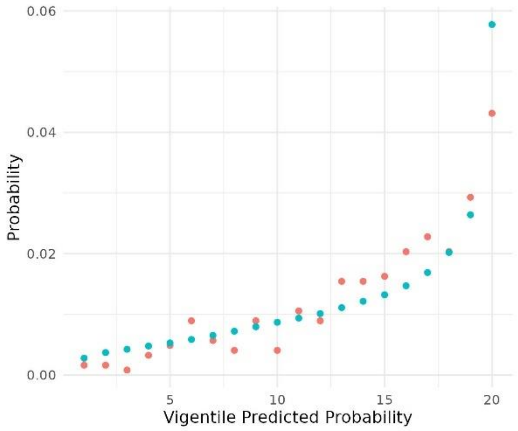

A. Wild-type

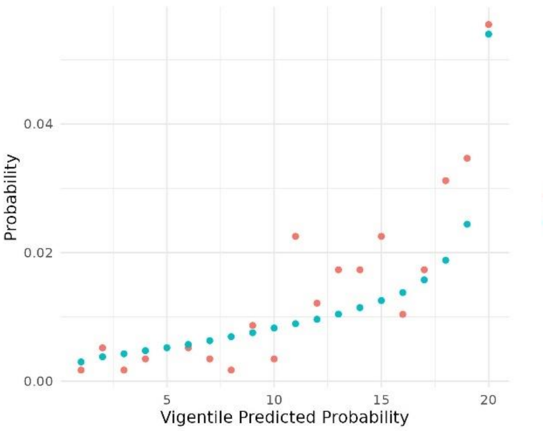

B. Alpha

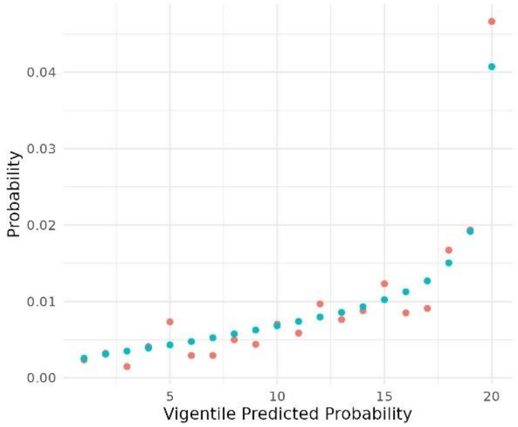

C. Delta

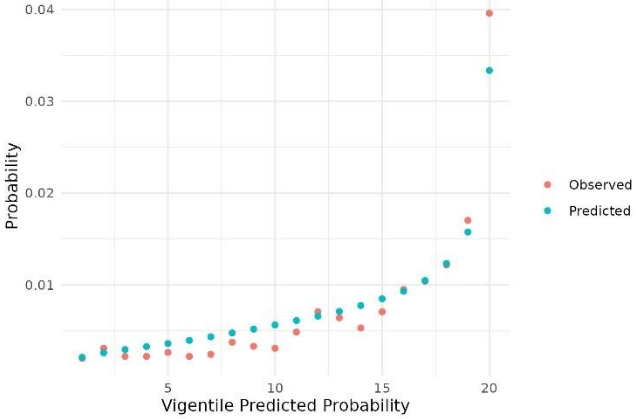

D. Omicron

Figure S5: Observed and predicted probabilities at each vigintile of predicted probabilities, by variant
The plot illustrates the observed and predicted probabilities of long COVID at each vigintile of predicted probability in our holdout dataset (N = 219,221), stratified by the dominant SARS-CoV-2 variant in the week of individuals positive RT-PCR tests. Panels A, B, C, and D present data for individuals who first tested positive while the Wild-type, Alpha, Delta, and Omicron variants were dominant (representing >60% of sequenced cases), respectively.

Sensitivity analyses

Incorporating data from individuals with positive LFTs

Although a positive RT-PCR test is generally accepted as the most reliable marker of COVID-19, not all individuals with COVID-19 received a positive RT-PCR test. As a sensitivity test, we repeated the main analysis using positive RT-PCR or positive LFT results to identify COVID-19 cases. This increased the number of individuals in the cohort to 1,458,018. A random 80:20 split resulted in testing and holdout datasets containing 1,166,414 and 291,604 individuals, respectively. The resultant model was substantively unchanged from the model derived using the main training dataset, with the exception that having received any number of doses of COVID-19 vaccination was found to be associated with reduced risk of developing long COVID (no significant association was observed in the main analysis) (Figure S6). Model performance evaluated in the holdout data was statistically indistinguishable from model performance in the main analysis (Table S9).

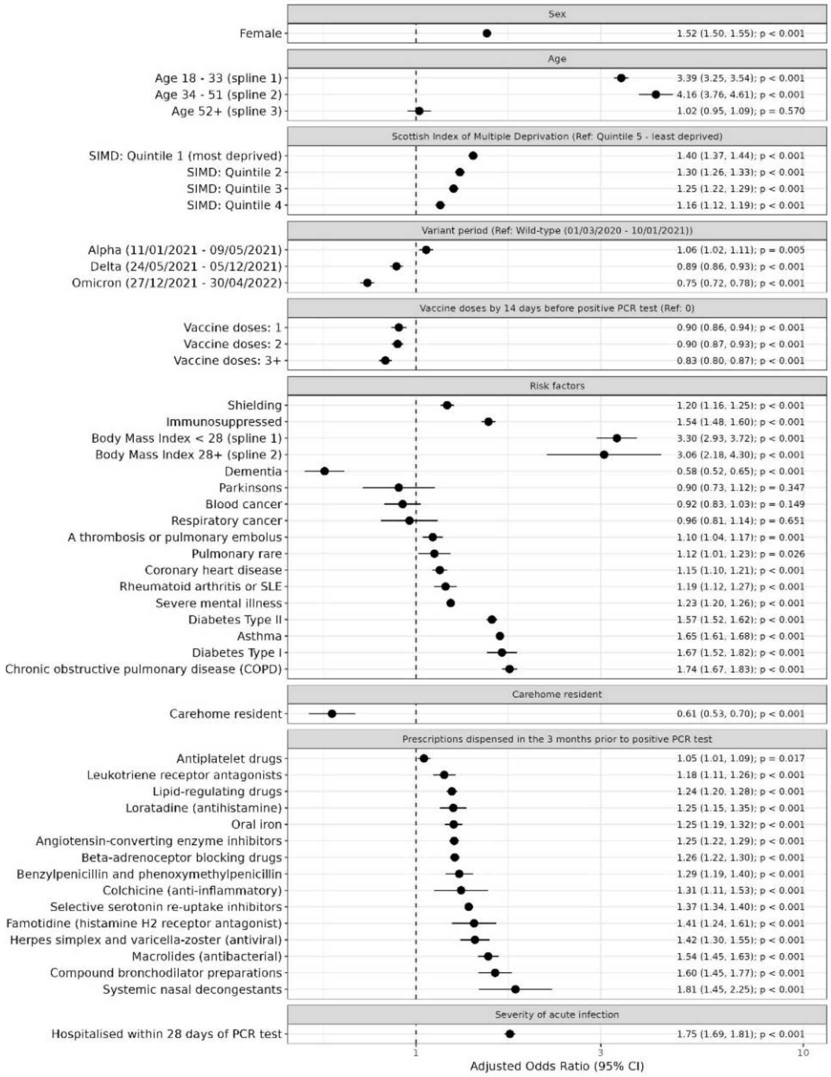

Figure S6: Adjusted odds ratios for predictors of long COVID estimated for all individuals with a positive RT-PCR or LFT result

The plot illustrates the adjusted odds ratios and 95% confidence intervals for all predictors of long COVID included in the main multivariable logistic regression model. The model was trained on a random 80% of a version of the cohort that included all individuals with a positive RT-PCR of LFT result (n=1,166,414) and fine-tuned using 10-fold cross-validation. SIMD quintiles relate to quintiles of the Scottish Index of Multiple Deprivation.

Table S9: Evaluation metrics (95% confidence interval) for the multivariable logistic regression model tested in holdout data, trained and tested on datasets containing (i) all individuals with a positive RT-PCR test result, and (ii) all individuals with a positive RT-PCR or LFT result

	**Main model**	**Positive RT-PCR or LFT model**
N	219,221	291,604
C-statistic	0.714 (0.710-0.719)	0.708 (0.704-0.712)
AUC precision-recall	0.136 (0.133-0.139)	0.135 (0.132-0.138)
Calibration slope	1.010 (0.986-1.034)	0.993 (0.972-1.014)
Discrimination threshold (equal to prevalence of the dependent variable)	*0.056*	*0.056*
Sensitivity (Recall)	0.651 (0.643-0.660)	0.635 (0.627-0.642)
Specificity	0.668 (0.666-0.670)	0.676 (0.674-0.678)
Accuracy	0.667 (0.665-0.669)	0.674 (0.672-0.675)
Positive predicted values (PPV)	0.104 (0.102-0.106)	0.104 (0.102-0.106)
Negative predicted values (NPV)	0.970 (0.969-0.971)	0.969 (0.968-0.970)
F1 Score	0.179 (0.178-0.181)	0.179 (0.177-0.180)
Matthew's correlation coefficient	0.154 (0.152-0.156)	0.151 (0.149-0.153)
Brier score	0.333 (0.331-0.335)	0.326 (0. 325-0.328)

Omitting individuals with incomplete follow-up

Some participants had fewer than 26 weeks of follow-up data after receiving a positive RT-PCR test, due to (i) censoring for death or reinfection, or (ii) testing positive fewer than 26 weeks before the study end date. To test the possibility that individuals with incomplete follow-up biased our results (by being less likely to be identified as having long COVID), we repeated the main analysis on a restricted subset of the training data that included only those participants with complete follow-up, retaining 94.1% of participants (n = 825,184 and n = 206,357, respectively). Training and evaluating the model on these restricted datasets produced a model with patterns of predictors that were generally consistent with the main model (Figure S7), with the exception that having received three doses of COVID-19 vaccination was associated with increased risk of developing long COVID. Evaluating the model in the restricted holdout dataset revealed consistency with the main model, with very marginal improvements in positive predicted values (PPV) and F1 score (Table S10).

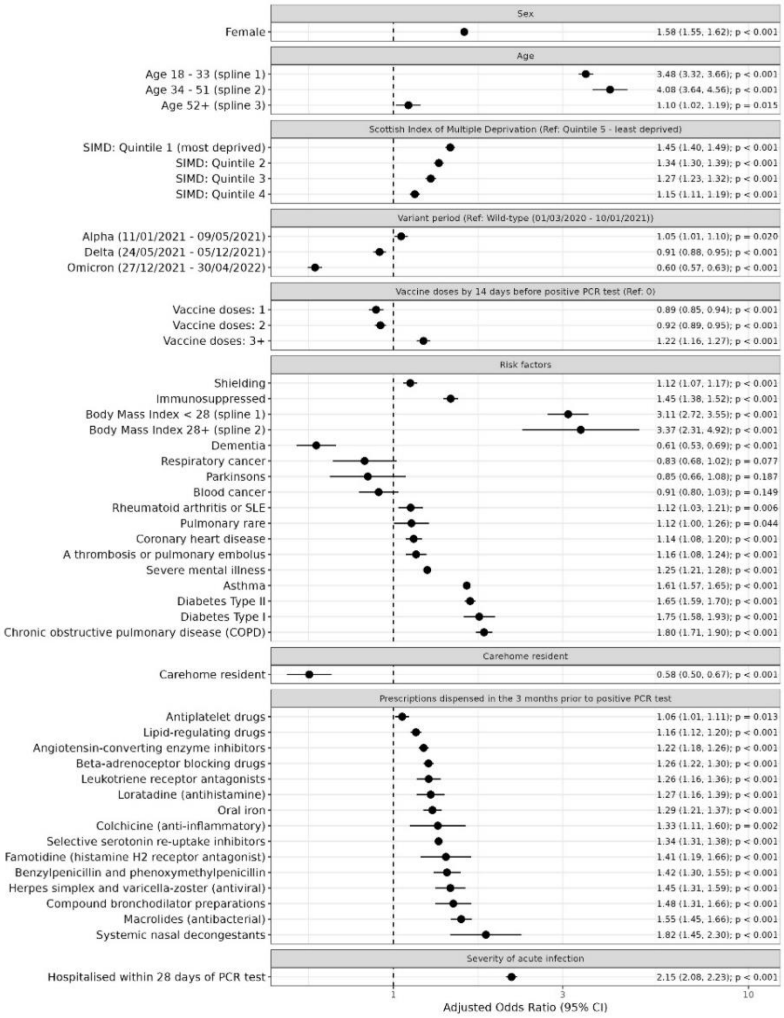

Figure S7: Adjusted odds ratios for predictors of long COVID for individuals with complete follow up

The plot presents adjusted odds ratios and their 95% confidence intervals for all predictors of long COVID included in our main multivariable logistic regression model. The model was trained on a restricted sample of the training dataset, containing individuals who had the full 26 weeks of follow up data (94.1% of the training dataset, n = 825,184). The model was fine-tuned using 10-fold cross-validation. SIMD quintiles relate to quintiles of the Scottish Index of Multiple Deprivation.

Table S10: Evaluation metrics (95% confidence interval) for the multivariable logistic regression model trained and tested on datasets containing individuals with complete follow up

	**Main model**	**Complete follow up model**
N	219,221	206,357
C-statistic	0.714 (0.710-0.719)	0.723 (0.719-0.727)
AUC precision-recall	0.136 (0.133-0.139)	0.160 (0.158-0.163)
Calibration slope	1.010 (0.986-1.034)	1.011 (0.988-1.034)
Discrimination threshold (equal to prevalence of the dependent variable)	*0.056*	*0.063*
Sensitivity (Recall)	0.651 (0.643-0.660)	0.721 (0.713-0.729)
Specificity	0.668 (0.666-0.670)	0.599 (0.597-0. 601)
Accuracy	0.667 (0.665-0.669)	0.607 (0.604-0.609)
Positive predicted values (PPV)	0.104 (0.102-0.106)	0. 108 (0.106-0.111)
Negative predicted values (NPV)	0.970 (0.969-0.971)	0.969 (0.968-0.970)
F1 Score	0.179 (0.178-0.181)	0.189 (0.187-0.190)
Matthew's correlation coefficient	0.154 (0.152-0.156)	0.158 (0.155-0.161)
Brier score	0.333 (0.331-0.335)	0.393 (0.391-0.395)

Variations of the main outcome measure

There is no "gold standard" approach to identifying cases of long COVID. Formal diagnoses of long COVID are recorded in EHRs at a considerably lower rate than estimated prevalence.[1,5] This may reflect delays in the availability of long COVID codes or terminology early in the pandemic, clinicians' lack of familiarity with the condition or codes, or hesitancy to code long COVID due to clinical uncertainty. Our outcome measure was designed to capture explicit diagnoses of long COVID, as well as probable cases where formal diagnoses had not been made. Explicit diagnoses of long COVID were identified using long COVID clinical codes and free text mentions of long COVID recorded in primary care EHRs or on sick notes issued in primary care. This was supplemented with an operational definition, which identified individuals with symptoms, investigations, and management strategies consistent with long COVID (identified through statistical analyses of EHRs) recorded in their EHRs - including where no explicit long COVID diagnosis had been made.[1]

However, the operational definition may misclassify cases that present similarly to long COVID. To investigate the possible impact of misclassification, we repeated the main analysis using two variations of the outcome measure. The first identified cases of long COVID using only long COVID clinical codes or free text recorded in primary care or on sick notes (i.e. omitting cases identified only by the operational definition). To address the possibility that the operational definition misclassified individuals with health conditions that require regular blood tests as having long COVID, we also used a version of the operational definition that did not include blood tests as a feature with which to identify cases of long COVID. The number of individuals classified as having long COVID according to each measure is presented in Table S11.

Table S11: Variations of the long COVID outcome measure

Outcome measure	Long COVID prevalence: training data	Long COVID prevalence: testing data
Operational definition, long COVID clinical code, free text, or sick note (main outcome measure)	48,888 (5.6%)	12,235 (5.6%)
Long COVID clinical code, free text, or sick note	12,675 (1.4%)	3,185 (1.4%)
Operational definition (excluding blood tests), long COVID clinical code, free text, or sick note	19,246 (2.2%)	4,782 (2.2%)

Irrespective of which outcome measure the model was trained on, the associations between long COVID and the sociodemographic predictors, as well as some of the clinical predictors, were consistent (Figure S8). All models identified a positive associated between long COVID and severe mental illness, coronary heart disease, and asthma. However, the model trained on the main outcome measure was the only one to identify positive associations between long COVID and: being advised to shield against COVID-19; being immunosuppressed; having Type 1 or Type 2 diabetes; rheumatoid arthritis or systemic lupus erythematosus; or a thrombosis or pulmonary embolus. Similarly, while the model trained on the main outcome measure identified positive associations between long COVID and 14 of the 15 prescriptions tested, the other models identified considerably fewer positive associations. Across all models, positive associations with long COVID were identified for just five types of prescriptions: beta-adrenoceptor blocking drugs, herpes simplex and varicella-zoster (antivirals), leukotriene receptor antagonists, selective serotonin re-uptake inhibitors, and macrolides (antibacterials).

The three models performed consistently in the holdout data in terms of c-statistics, ratios between AUC precision-recall, and calibration slope (Table S12). When evaluated in holdout data at a discrimination threshold set equal to the discrimination threshold used to evaluate the main model (0.056), the additional models' performance was statistically indistinguishable from the main model in terms of sensitivity, specificity, accuracy, and Brier score. However, both models performed worse than the main model in terms of Positive Predicted Values (PPV) and F1 Score, indicating a tendency to produce more false positives.

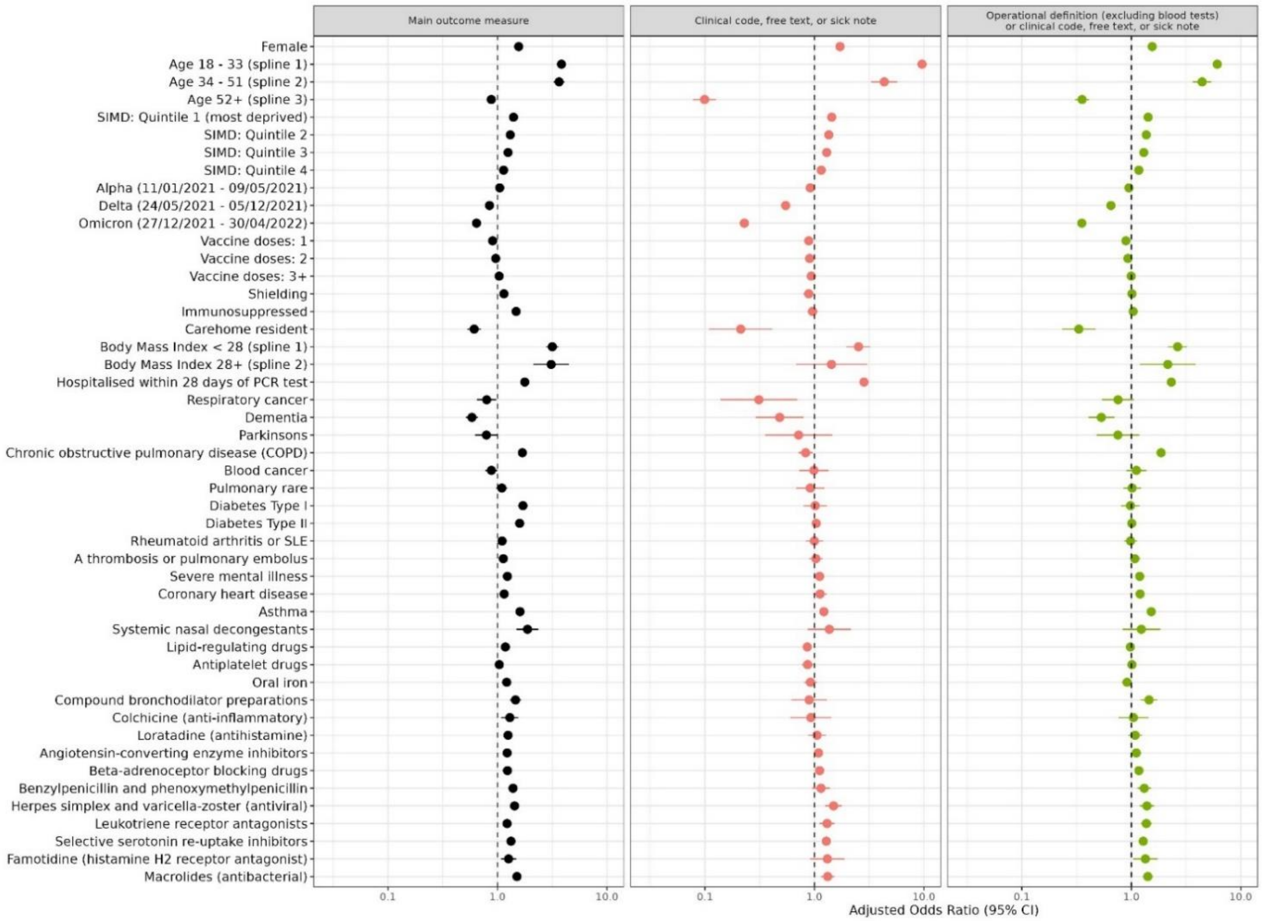

Figure S8: Adjusted odds ratios for predictors of long COVID estimated using alternative outcome measures
The plot illustrates the adjusted odds ratios and their 95% confidence intervals for all predictors of long COVID, estimated using multivariable logistic regression. Each panel presents results estimated using a different outcome measure. Reference categories are: Sex: Male; SIMD quintile: 5; Variant period: Wild (up to 10/01/2021); Vaccine doses: 0. The model was trained on the training dataset (N = 876,885) with 10-fold cross-validation. Prevalence of long COVID identified by each outcome measure was: 5.6% (N = 48,888) when using the main outcome measure, 1.4% (N = 12,675) when using clinical, free text, or sick notes; and 2.2% (N = 19,246) when using the main outcome measure, but excluding blood tests from the operational definition. SIMD quintiles relate to quintiles of the Scottish Index of Multiple Deprivation.

Table S12: Evaluation metrics (95% confidence interval) for the multivariable logistic regression model in the holdout dataset (n = 219,221), for variations on the outcome measure

	Main model (operational definition, long COVID clinical code, free text, or sick note)	Long COVID clinical code, free text, or sick note	Operational definition (excluding blood tests), long COVID clinical code, free text, or sick note
C-statistic	0.714 (0.710-0.719)	0.760 (0.752-0.768)	0.730 (0.722-0.737)
AUC precision-recall	0.136 (0.133-0.139)	0.035 (0.032-0.037)	0.065 (0.061-0.069)
Calibration slope	1.010 (0.986-1.034)	1.009 (0.971-1.046)	1.017 (0.967-1.066)
Discrimination threshold (equal to prevalence of the main outcome measure)	*0.056*	*0.056*	*0.056*
Observed prevalence of outcome measure	0.056	0.014	0.022
Sensitivity (Recall)	0.651 (0.643-0.660)	0.693 (0.677-0.709)	0.680 (0.667-0.693)
Specificity	0.668 (0.666-0.670)	0.691 (0.689-0.693)	0.661 (0.659-0.663)
Accuracy	0.667 (0.665-0.669)	0.691 (0.689-0.693)	0.661 (0.659-0.663)
Positive predicted values (PPV)	0.104 (0.102-0.106)	0.032 (0.031-0.033)	0.043 (0.041-0.044)
Negative predicted values (NPV)	0.970 (0.969-0.971)	0.993 (0.993-0.994)	0.989 (0.989-0.990)
F1 Score	0.179 (0.178-0.181)	0.061 (0.060-0.062)	0.081 (0.079-0.082)
Matthew’s correlation coefficient	0.154 (0.152-0.156)	0.099 (0.098-0.099)	0.105 (0.103-0.107)
Brier score	0.333 (0.331-0.335)	0.309 (0.307-0.311)	0.339 (0.337-0.341)

The table presents evaluation metrics for the main model, trained using logistic regression with 10-fold cross validation in the training dataset (N = 876,885) and evaluated in the holdout dataset (N = 219,221). The model was trained using the main outcome measure (Operational definition, long COVID clinical code, free text, or sick note), and performance was evaluated when using each of the outcome measures indicated in the column headings.

Models derived using machine learning methods
We compared the performance of the logistic regression model to a Naïve Bayes Classifier model (including a Laplace correction equal to 1 to reduce overfitting) and a gradient boosted decision tree model (using the XGBoost algorithm) with 10-fold cross validation to identify the optimal number of model iterations. Feature importance scores from the XGBoost model (Figure S9) identified variant period, age, severity of acute infection, sex, vaccine doses, BMI, and deprivation among the most important predictors of long COVID, in general alignment with the results of our main analysis.

The logistic regression model and the Naïve Bayes Classifier model performed similarly in terms of the c-statistic and AUC precision-recall, while the XGBoost model performed less well (Table S13). When evaluated at a discrimination threshold set equal to the observed prevalence of long COVID, the logistic regression model performed better than the other two models in terms of balance between correctly identifying true positives and false negatives. Compared with the main model, both machine learning models were more conservative in their identification of positive cases (lower sensitivity), but a higher share of their positive predictions were correct (higher PPV).

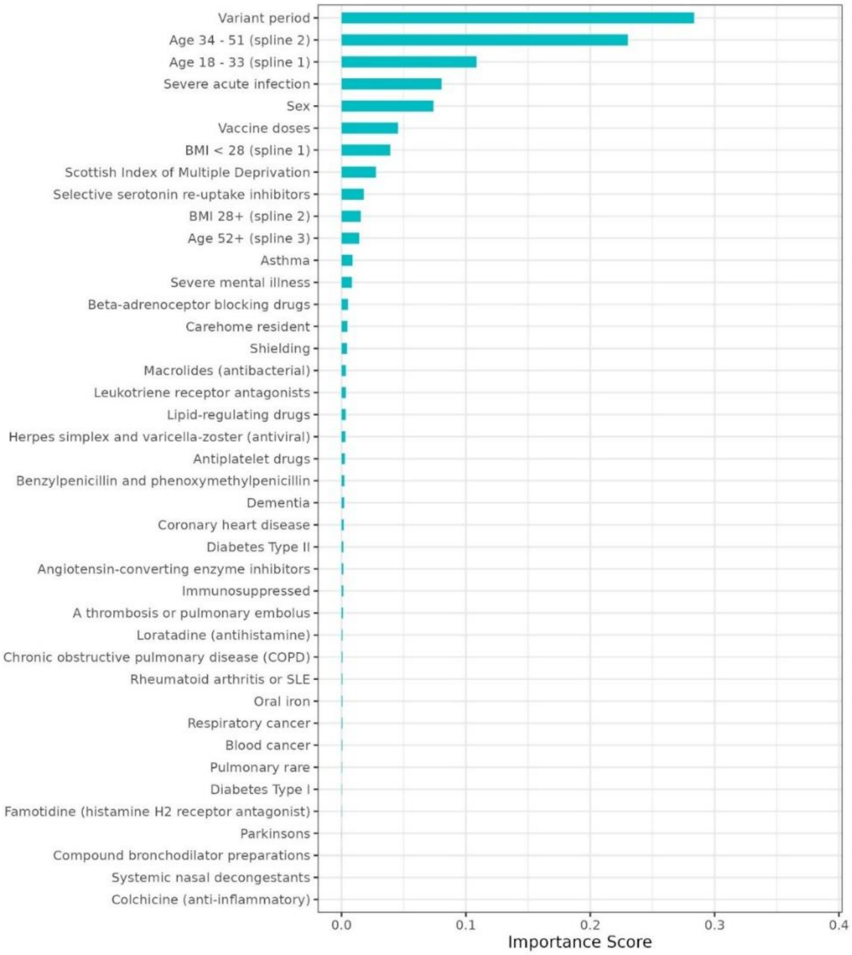

Figure S9: Feature importance scores estimated using a Gradient Boosted Decision Tree
The plot illustrates feature importance scores for our main model, estimated using the Gradient Boosted Decision Tree algorithm, XGBoost. 10-fold cross validation was used to identify the optimal number of iterations (N = 51). The algorithm was trained on the training dataset (N = 876,885).

Table S13: Evaluation metrics for models trained using multivariable logistic regression (main model), XGBoost, and a Naïve Bayes Classifier

	Main model performance in holdout data	XGBoost	Naïve Bayes Classifier
C-statistic	0.714 (0.710-0.719)	0.642 (0.636-0.647)	0.693 (0.691-0.695)
AUC precision-recall	0.136 (0.133-0.139)	0.101 (0.099-0.103)	0.151 (0.148-0.154)
Calibration slope	1.010 (0.986-1.034)	0.536 (0.517-0.555)	1.148 (1.114-1.185)
Discrimination threshold (equal to prevalence of the outcome measure)	*0.056*	*0.056*	*0.056*
Sensitivity	0.651 (0.643-0.660)	0.095 (0.090-0.100)	0.351 (0.347-0.356)
Specificity	0.668 (0.666-0.670)	0.971 (0.970-0.971)	0.848 (0.847-0.849)
Accuracy	0.667 (0.665-0.669)	0.922 (0.921-0.923)	0.820 (0.819-0.821)
Positive predicted values	0.104 (0.102-0.106)	0.160 (0.151-0.168)	0.120 (0.118-0.122)
Negative predicted values	0.970 (0.969-0.971)	0.948 (0.947-0.949)	0.957 (0.956-0.957)
F1 Score	0.179 (0.178-0.181)	0.119 (0.117-0.120)	0.179 (0.178-0.180)
Matthew's correlation coefficient	0.154 (0.152-0.156)	0.084 (0.082-0.086)	0.124 (0.120-0.128)
Brier score	0.333 (0.331-0.335)	0.078 (0.077-0.079)	0.180 (0.179-0.181)

The table presents evaluation metrics for models trained using the approach indicated in the column header. Models were trained in the training dataset (N = 876,885) and performance was evaluated in the holdout dataset (N = 219,221).

Model training and testing using a geographic split

To assess the generalisability of our approach we re-trained the model using a training dataset that contained data for patients registered with a GP in 12 of Scotland's 14 health boards (regional authorities with responsibility for the delivery of health services, representing 70% of the cohort (n = 767,753)), and tested the model in each of two holdout regions: Lothian (15.6% of the cohort, n = 170,752) and Lanarkshire (14.4% of the cohort, n = 157,601). Observed prevalence of long COVID was 3.5% (N = 6,039) in Lothian, and 5.2% (N = 8,258) in Lanarkshire. The model was trained as before, using multivariable logistic regression with 10-fold cross validation. The model derived closely resembled the main model (Figure S10).

Table S14 presents statistics evaluating model performance in each of the two holdout regions, evaluated at a discrimination threshold equal to the observed prevalence of long COVID in the main dataset (0.056). In each holdout region, the c-statistic and calibration slope were statistically indistinguishable from the main analysis. Model performance in the Lothian holdout dataset was generally consistent with the main analysis, but marginally worse in terms of the model's ability to accurately predict positive cases (indicated by lower PPV and F1 Score). There was more variation in model performance when assessed in the Lanarkshire holdout dataset, where the model correctly identified more true positives (higher sensitivity), but fewer true negatives (lower specificity).

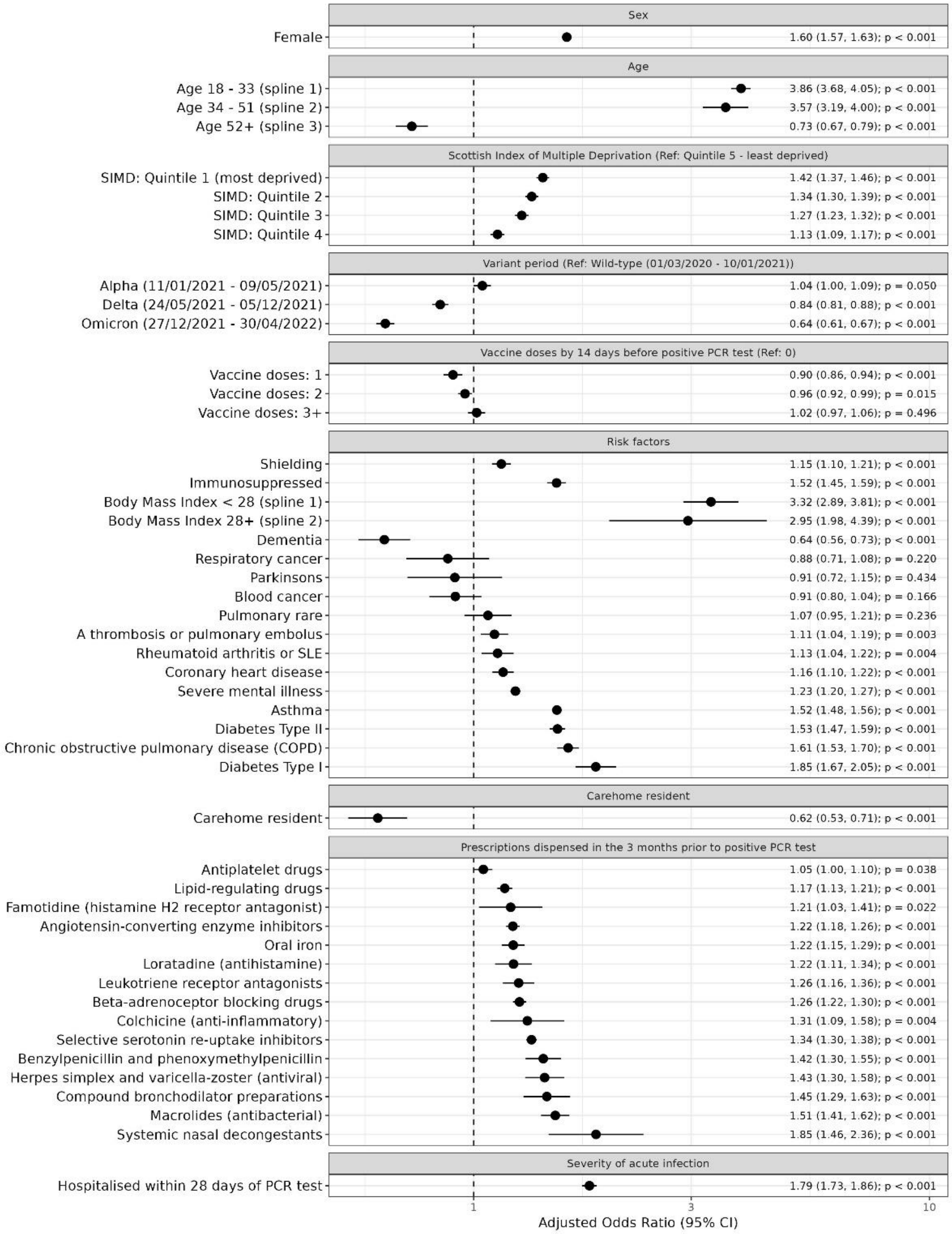

Figure S10: Adjusted odds ratios for predictors of long COVID, trained in 12 of Scotland's 14 health boards The plot illustrates the adjusted odds ratios and 95% confidence intervals for all predictors of long COVID included in the main multivariable logistic regression model. This version of the model was trained on data for individuals registered with GPs in 12 of Scotland 14 health boards (n=767,753) using multivariable logistic regression with 10-fold cross-validation. SIMD quintiles relate to quintiles of the Scottish Index of Multiple Deprivation.

Table S14: Evaluation metrics for the main multivariable logistic regression model compared and models trained and tested using a geographic split

	Main model performance in holdout data	Lanarkshire (14.4%) trained on 12 regions	Lothian (15.6%) trained on 12 regions
C-statistic	0.714 (0.710-0.719)	0.717 (0.711 - 0.722)	0.713 (0.706 - 0.719)
AUC precision-recall	0.136 (0.133-0.139)	0.125 (0.121-0.129)	0.094 (0.091-0.97)
Calibration slope	1.010 (0.986-1.034)	1.023 (0.994-1.053)	1.051 (1.017-1.085)
Discrimination threshold (equal to prevalence of the outcome measure)	*0.056*	*0.056*	*0.056*
Observed prevalence	0.056	0.052	0.035
Sensitivity	0.651 (0.643-0.660)	0.746 (0.736-0.755)	0.658 (0.646-0.670)
Specificity	0.668 (0.666-0.670)	0.565 (0.562-0.567)	0.650 (0.648-0.653)
Accuracy	0.667 (0.665-0.669)	0.574 (0.572-0.577)	0.650 (0.648-0.653)
Positive predicted values	0.104 (0.102-0.106)	0.087 (0.084-0.089)	0.065 (0.063-0.066)
Negative predicted values	0.970 (0.969-0.971)	0.976 (0.975-0.977)	0.981 (0.980-0.982)
F1 Score	0.179 (0.178-0.181)	0.155 (0.153-0.157)	0.118 (0.116-0.119)
Matthew's correlation coefficient	0.154 (0.152-0.156)	0.143 (0.141-0.158)	0.124 (0.125-0.123)
Brier score	0.333 (0.331-0.335)	0.426 (0.423-0.428)	0.350 (0.347-0.352)

The table presents evaluation metrics for model performance in holdout data for the model trained and tested using a random 80:20 split, and for models trained on data for individuals registered with GPs in 12 of Scotland 14 health boards (n=767,753) and tested in two holdout regions. All models were trained using multivariable logistic regression with 10-fold cross-validation. All models were evaluated using a discrimination threshold set equal to prevalence of long COVID observed in the main training and testing datasets (0.056).

References

[1] Jeffrey K, Woolford L, Maini R et al. Prevalence and risk factors for long COVID among adults in Scotland using electronic health records: a national, retrospective, observational cohort study. *EClinicalMedicine* 2024;71:102590

[2] Clift AK, Coupland CAC, Keogh RH, et al. Living risk prediction algorithm (QCOVID) for risk of hospital admission and mortality from coronavirus 19 in adults: national derivation and validation cohort study. BMJ 2020;371:m3731.

[3] University of Edinburgh. Our Public Advisory Group (PAG). https://www.ed.ac.uk/usher/eave-ii/meet-researchers-public-advisors/eave-ii-public-advisory-group (Accessed 13th May 2024)

[4] NIHR. Payment guidance for researchers and professionals. https://www.nihr.ac.uk/documents/payment-guidance-for-researchers-and-professionals/27392#payment-rates (Accessed 13th May 2024)

[5] Walker AJ, MacKenna B, Inglesby P, et al. Clinical coding of long COVID in English primary care: a federated analysis of 58 million patient records in situ using OpenSAFELY. Br J Gen Pract 2021;71(712):e806–14.
